# Nanopore sequencing of DNA-barcoded probes for highly multiplexed detection of microRNA, proteins and small biomarkers

**DOI:** 10.1038/s41565-023-01479-z

**Published:** 2023-09-25

**Authors:** Caroline Koch, Benedict Reilly-O’Donnell, Richard Gutierrez, Carla Lucarelli, Fu Siong Ng, Julia Gorelik, Aleksandar P. Ivanov, Joshua B. Edel

**Affiliations:** 1 Department of Chemistry, Molecular Science Research Hub, Imperial College London, London, UK; 2 National Heart and Lung Institute, ICTEM, Imperial College London, London, UK; 3 Oxford Nanopore Technologies PLC, Oxford Science Park, Oxford, UK

**Keywords:** Nanopores, Biosensors

## Abstract

There is an unmet need to develop low-cost, rapid and highly multiplexed diagnostic technology platforms for quantitatively detecting blood biomarkers to advance clinical diagnostics beyond the single biomarker model. Here we perform nanopore sequencing of DNA-barcoded molecular probes engineered to recognize a panel of analytes. This allows for highly multiplexed and simultaneous quantitative detection of at least 40 targets, such as microRNAs, proteins and neurotransmitters, on the basis of the translocation dynamics of each probe as it passes through a nanopore. Our workflow is built around a commercially available MinION sequencing device, offering a one-hour turnaround time from sample preparation to results. We also demonstrate that the strategy can directly detect cardiovascular disease-associated microRNA from human serum without extraction or amplification. Due to the modularity of barcoded probes, the number and type of targets detected can be significantly expanded.

## Abstract (web summary)

This study establishes a method for multiplexed detection of up to 40 different biomarkers in a single assay by combining nanopore sequencing with analyte-selective barcoded probes, including cardiac disease-associated microRNA directly from blood serum.

## Main

The detection of blood serum biomarkers is one of the most common methods for diagnosis, prognosis, predicting future disease and monitoring response to treatment. Biochemicals have been used for over 60 years to aid diagnosis of a wide range of conditions, including cancer^1^, pregnancy^2^ and cardiac disease^3^. Biomarker tests traditionally rely on the detection of proteins to indicate a condition. However, these tests often lack the specificity to provide clinically useful detail on the pathology^4,5^. To increase the specificity in detecting diseases, multiplexed biomarker assays have been developed. Such strategies have been attempted in several areas, including Alzheimer’s disease^6,7^, amyotrophic lateral sclerosis^8^, cardiovascular disease^9–11^, chronic obstructive pulmonary disease^12^, infection^13^ and cancers^14–17^. Many of the methodologies employed rely upon antibody recognition of an epitope and an associated optical readout for each analyte, for example, ELISA, which can be sensitive to pM levels of analyte. Commercially available systems can detect up to 80 proteins simultaneously^18,19^. Other methods use modified DNA-based aptamers, which allow the detection of multiple proteins in a single sample with a sensitivity down to 125 fM (refs. ^20,21^). These techniques offer promising tools for high-throughput protein biomarker detection but are unable to observe multiple molecular species simultaneously.

Clinical diagnostics are now moving beyond protein biomarkers and genetic testing. One example is microRNAs (miRNAs), short non-coding RNAs that regulate gene expression^22,23^. Alterations in miRNA expression have been identified in a wide range of clinical areas, such as cardiology^24^, hepatology^25^, nephrology^26^, neurology^27^, oncology^28^ and vascular disease^29^. These molecules are detected in the blood primarily through reverse transcription–quantitative PCR (RT–qPCR)^30^, which is sensitive down to fM. However, this method requires multiple steps and relies on signal amplification, which may introduce bias in the measurement. Furthermore, the rapid degradation of miRNAs provides a particular problem when considering their use in a clinical setting. A rapid miRNA profiling platform would offer the potential to capture short-lived events and perform frequent longitudinal testing.

There is, therefore, a great need to develop technologies that can perform highly multiplexed detection of various analyte classes, including nucleic acids, proteins and small molecules. Single-molecule nanopore sensing offers the ideal platform for performing this task. Nanopores have previously been shown to enable efficient detection of DNA, RNA, proteins and other molecules^31–35^, albeit not in a highly multiplexed configuration. Analyte detection by nanopores depends on measuring current fluctuations as charged molecules are electrophoretically driven through a nanoscale aperture. The translocation of a molecule through a nanopore causes a change in the ionic current, which is dependent on the molecule’s charge, size and conformation^36^. However, the method generally lacks selectivity. Strategies to address these limitations include chemical modifications of the pore^37–40^, the use of molecular carriers^41^ and the use of electro-optical methods^42,43^. These methods are excellent for the detection of molecules at very low concentrations without amplification; however, their throughput and multiplexing ability are limited.

Biological nanopores are advantageous over solid-state devices as they are highly reproducible and can be engineered for specific functionality^44^. In particular, biological nanopores are useful for DNA/RNA sequencing, as shown by Oxford Nanopore Technologies (ONT). ONT has commercialized sequencing devices that use biological nanopore arrays to allow for high-throughput, simultaneous DNA/RNA reads^45^ of fragments ≥20 base pairs^46^.

This study showcases a multiplexed analyte detection strategy, combining nanopore sequencing with DNA-barcoded molecular probes. The platform allows accurate demultiplexing of events, with simultaneous quantitative detection of at least 40 molecules that can consist of miRNAs, proteins and small molecules, such as neurotransmitters. The presence of each analyte is determined by the translocation dynamics of each probe as it passes through a nanopore. In this study, we selected 40 miRNAs and proteins implicated in cardiac disease. The method established is easily adaptable and scalable, meaning the number of detected biomarkers can be extended to cover multiple diseases. The assay requires a sample volume of less than 30 μl, does not require sample labelling or amplification and costs less than US$100. Furthermore, the technology bears the potential for pooled patient analysis, which could further reduce the cost per test. Due to the simplicity of the experimental protocol, the portability of the platform and the rapid turnaround time for experiments, we are confident that this approach could have an extensive impact on current diagnostics.

## Strategy for the multiplexed detection of analytes

**Fig. 1:**
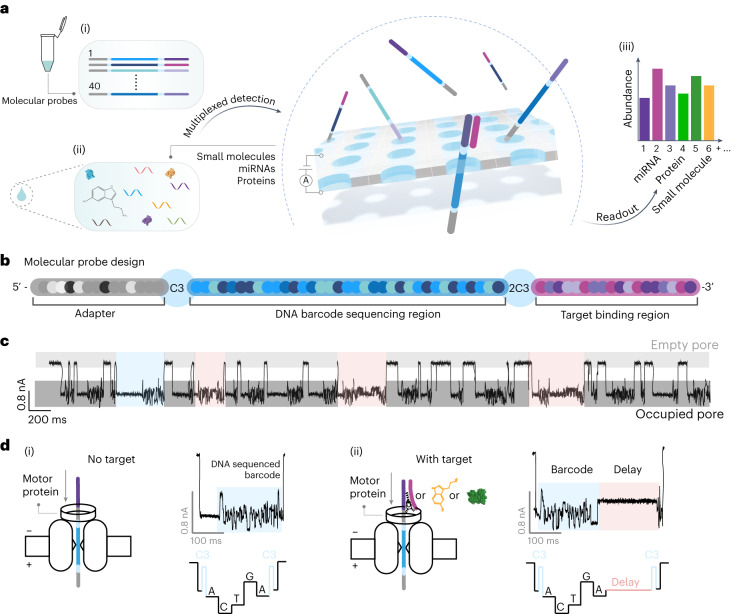
Demultiplexing of 40 miRNAs, proteins and small molecules using barcoded probes and nanopore sequencing. **a**, Workflow for detection of miRNAs, proteins and small molecules. Barcoded probes (i) were incubated with synthetic targets or serum samples from healthy participants (ii). Nanopore sequencing was performed to classify the barcode, and the presence of target analytes (iii) was determined. **b**, Barcoded probe design. The probe consists of a Y-adapter containing the motor protein; the barcode region, containing 35 bases; and the target binding region at the 5′ end of the probe, which includes either a complementary sequence to a miRNA or an aptamer designed to specifically bind a protein or small molecule. **c**, Characteristic current traces. Example events are highlighted for barcoded probe only and barcoded probe events with bound target analytes. **d**, Base calling and analysis of events from barcoded probes result in current traces without delay (i). Base calling and analysis of events from barcoded probes with analyte bound results in current traces with a delay (ii).

A highly multiplexed detection strategy was achieved by combining nanopore sequencing with barcoded molecular probes that selectively bind to target analytes (Fig. 1a and Supplementary Table 1). Barcoded probes were incubated with target analytes (miRNAs, proteins, small molecules), sequenced with the MinION device (ONT) and, subsequently, the presence of target analytes was determined. The barcoded probes consist of three key regions: (1) adapter; (2) barcode; and (3) target binding region (Fig. 1b). The adapter is identical for all probes. The barcode sequence acts as a unique identifier and can have many permutations, with theoretically up to 1.18 × 10^21^ distinctive arrangements of the nucleotides for the barcode length used in these experiments. The target binding region can either be a complementary sequence (to bind miRNA or DNA) or an aptamer (to bind proteins and small molecules). The translocation of hybridized probes (target is bound) is slowed since the nanopore geometry does not allow the passage of a double-strand or protein/small molecule to feed through the nanopore (Fig. 1c). The slowed translocation can be observed in the event current signal as a ‘delay’ period. Events can then be subclassified with or without delay (Fig. 1d), revealing at the single-molecule level the presence of an analyte (Supplementary Fig. 1), whilst the sequenced barcode classifies the analyte being targeted.

**Fig. 2:**
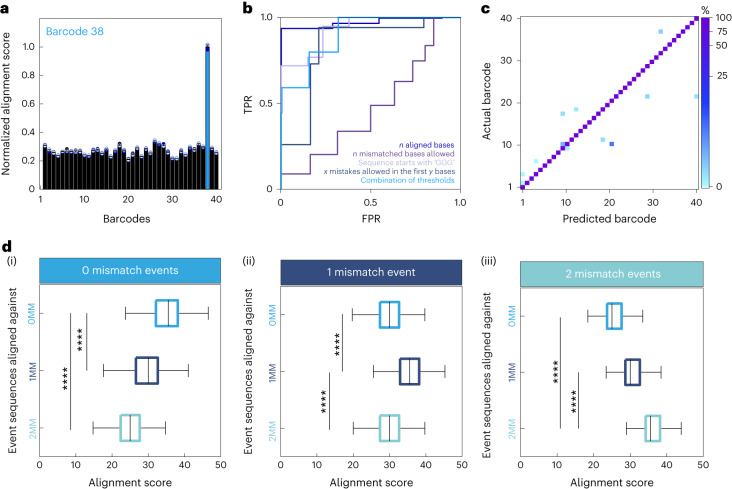
Highly accurate detection of nucleic acid barcodes. **a**, Alignment scores of a barcoded probe 38 experiment. Sequenced events were aligned against a barcode library containing all 40 barcoded probe sequences. The alignment score for barcoded probe 38 was significantly higher (analysis of variance (ANOVA), *F* (39, 17,360) = [3,441.41], ****P = 0, *n* = 3, *n*_total events_ = 454) than all other alignment scores observed. Data presented as mean ± s.d. **b**, Receiver operating characteristic curve showing the FPR and true-positive rate (TPR) of various alignment thresholds including *n* aligned bases; *n* mismatched bases allowed; sequence starts with *x*; *x* mismatches allowed in first *y* bases and a combination (*n* = 3). **c**, Confusion matrix of alignment accuracy of single barcoded probe experiments. Accuracy was >95% for all 40 barcoded probes tested (*n* = 3, *n*_total events_ = 15,489). **d**, Alignment score of barcoded probe sequences with 1 and 2 mismatched bases. A multiplexed experiment containing three barcoded probes (‘0MM’, GGGTGCACGAGTGCGTGT; ‘1MM’ GGGT**A**CACGAGTGCGTGT; ‘2MM’ GGGT**A**CA**T**GAGTGCGTGT) shows a significant difference between alignment scores. 0MM events had the highest alignment score for the 0MM sequence (ANOVA, *F* (2, 67,524) = [13,657.25], ****P = 0, *n* = 4, *n*_total events_ = 22,509) (i). 1MM events had the highest alignment score for the 1MM sequence (ANOVA, *F* (2, 33,204) = [2,702.66], ****P = 0, *n* = 4, *n*_total events_ = 11,069) (ii). 2MM events had the highest alignment score for the 2MM sequence (ANOVA, *F* (2, 33,597) = [7,763.95], ****P = 0, *n* = 4, *n*_total events_ = 11,200) (iii). Summary statistics for box plots: centre, median; bounds of box, interquartile range (IQR) 25th and 75th percentile; whiskers, minimum and maximum within 1.5 IQR. Barcoded probe concentration was 30 nM in all conditions. All experiments were performed in 2× sequencing buffer (700 mM KCl, 50 mM HEPES, 100 mM MgCl_2_, 100 mM ATP, 4.4 mM EDTA (pH 8.0)). Source data

To establish the multiplexed platform, we designed 40 unique barcoded probes to detect miRNAs implicated in cardiac disease. The accuracy of barcode base calling and classification was determined in experiments without target analytes (Fig. 2). Events were sequenced and aligned against a barcode library with known barcode sequences (Supplementary Table 1). For example, the alignment score of a unique barcode, ‘barcode 38’, is shown in Fig. 2a. The normalized alignment score of events for the true sequence was significantly higher compared to all other barcode sequences in the library (Fig. 2a, Supplementary Fig. 2a and Supplementary Table 2). However, alignment scoring was not suitable in multiplexed experiments due to the occurrence of false positives. To address this, a series of thresholds were tested, including: (1) the number of mismatches; (2) *x* mismatches in the first *y* bases; (3) number of aligned bases; and (4) sequence beginning with the bases ‘GGG’ (Fig. 2b). It was found that reducing the total number of mismatched bases worked poorly on its own (area under the curve (AUC) = 0.414). We observed that mismatches at the start of the sequenced event were particularly indicative of poorly resolved events. Consequently, a threshold based on allowing *x* mismatches in the first *y* bases was found to be moderately effective in removing unwanted events (AUC = 0.735). Increasing the total number of aligned bases was the second-best applied threshold (AUC = 0.828). Requiring the sequenced events to start with G residues (as this is common among all barcoded probes) proved to be the most efficient at separating true and false events (AUC = 0.846). Finally, we combined each criterion to determine the optimum threshold: ≤5 mismatched bases total; 1 mismatch in the first 10 bases; ≥15 bases aligned in total; sequence starts with ‘GGG’ (AUC = 0.805). This configuration did not improve sensitivity but markedly reduced the false-positive rate (FPR). The optimized thresholds resulted in an accuracy of >95% in the alignment of base-called events for single barcoded probe experiments (Fig. 2c). To determine the sensitivity of the platform, we tested a barcode against two further probes with one and two mismatched bases. In a multiplexed experiment with all three of the barcoded probes present, the alignment score for the correct barcode was significantly higher than the alignment scores of the other sequences (Fig. 2d).

## Hybridization of barcoded probes with miRNA

**Fig. 3:**
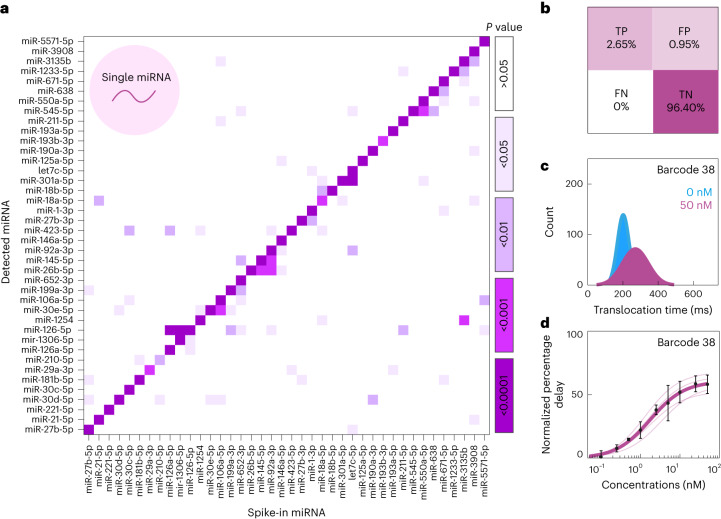
Sensitivity and selectivity of single miRNA detection. **a**, Heat map showing significance for selective miRNA binding. The 40 barcoded probes (30 nM) were incubated with each miRNA (10 nM), respectively. A significant increase (two-tailed *t*-test, **P ≤ 0.01) in percentage delayed events was observed for all barcoded probes when in the presence of their miRNA target (*n* = 3, *n*_total events_ = 712,400). **b**, Confusion matrix showing true positive (TP), false positive (FP), false negative (FN) and true negative (TN) occurrence. The accuracy was 99.05%, specificity was 99.02% and sensitivity was 100%. **c**, Translocation time of single barcoded probe and single miRNA experiments (*n* = 5, *n*_total events_ = 54,197). **d**, Concentration–percentage delay relationship of single miRNA and single barcoded probe experiment (barcoded probe 38, miR-221-5p). Data were fitted using the Hill equation with *n*_H_ = 1 (dissociation constant (*K*_d_)= 1.77 nM and *V*_max_ = 61.03%, *R*^2^ = 96.25, *n* = 5, *n*_total events_ = 54,197). Data presented as mean ± s.d. All experiments were performed in sequencing buffer. Source data

Many miRNAs share similar sequences^47^. To demonstrate sequence-specific miRNA detection, a mixture of 40 barcoded probes (30 nM) was incubated with each miRNA (10 nM) individually (Fig. 3a). It was found that the percentage delayed events of each barcoded probe increased significantly (*P* < 0.01) when in the presence of its corresponding target miRNA. We assessed the increase in percentage delayed events by comparing the miRNA sequence homologies (Supplementary Fig. 3a). Using a threshold of ≥90% miRNA sequence similarity (Supplementary Fig. 4a) and a significant increase in percentage delay (*P* ≤ 0.01), we found that 2.65% of all classifications were true positive, 0.95% false positive, 0% false negative and 96.40% true negative (Fig. 3b), resulting in a platform accuracy of 99.05%, specificity of 99.02% and sensitivity of 100%. Importantly, we observed that the barcode sequence does not influence the percentage delayed events detected for our probes (Supplementary Fig. 4b). In single barcoded probe experiments (for example, barcoded probe 38 and miR-221-5p), the total event time increased when 50 nM of miRNA was present compared to the control (Fig. 3c and Supplementary Fig. 2b). However, it is known that the DNA-controlling motor enzyme does not move the probe through the pore at a consistent rate; therefore, the speed at which the barcoded probes are translocated varies^48^. Hence, rather than relying on the translocation time, the moving standard deviation of the electrical signal was used to identify signal delays. Using this method, it was possible to determine a concentration–percentage delay curve for each barcoded probe and miRNA combination (*R*^2^ ≥ 0.989) (Fig. 3d and Supplementary Fig. 5).

## Multiplexed detection of miRNA, protein and small molecules

**Fig. 4:**
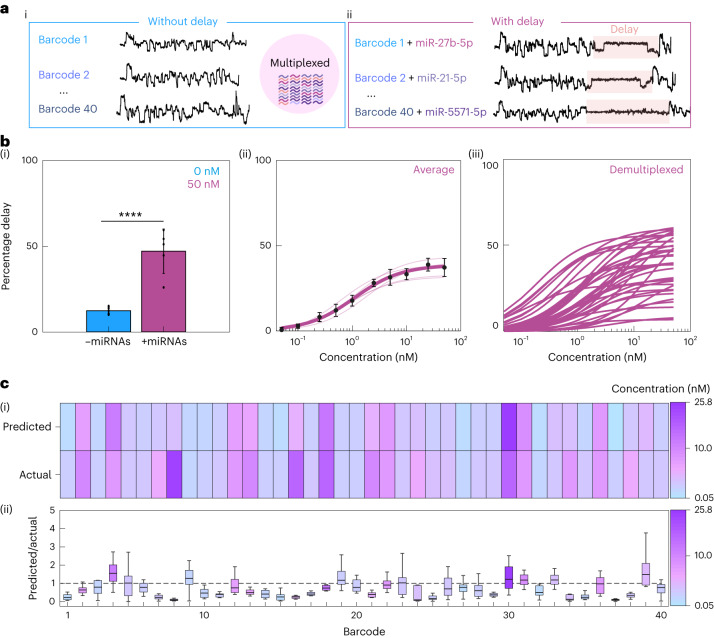
Multiplexed detection and quantification of miRNAs. **a**, Example current traces for barcodes without delay (i) and with delay (ii). **b**, Mean percentage delay ±50 nM miRNAs (two-tailed *t*-test, ****P = 3.58 × 10^−4^, *n* = 5, *n*_total events_ = 76,841 (0 nM), 69,983 (50 nM)). Data presented as mean ± s.d. (i). Multiplexed concentration–percentage delay curves of 40 barcoded probes (30 nM) and the corresponding 40 miRNAs. A curve was fitted using the Hill equation with *n*_H_ = 1 (*K*_d_ = 1.09 nM, *V*_max_ = 38.69%, *n* = 5, *n*_total events_ = 1,045,841). Data presented as mean ± s.d. (ii). Concentration–percentage delay curves of 40 individual barcoded probes derived from a multiplexed experiment. Curves were fitted with the Hill fit (*n*_H_ = 1) background subtraction for each curve was performed using the percentage delay value at 0 nM for each curve (*n* = 5, *n*_total events_ = 1,045,841) (iii). **c**, Single-blinded prediction of miRNA concentration and comparison to known concentration. Heat map of concentrations (*n* = 12, *n*_total events_ = 203,812) (i). Analysis of miRNA predictions showing the value for predicted/actual concentration (*n* = 12, *n*_total events_ = 203,812) (ii). Summary statistics for box plot: centre, median; bounds of box, IQR 25th and 75th percentile; whiskers, minimum and maximum within 1.5 IQR. All experiments were performed in sequencing buffer. Source data

Barcoded probes were incubated with synthetic miRNAs to identify whether event delays could be observed in multiplexed conditions (Fig. 4a). It was found that the mean percentage delayed events increased when miRNAs were present (0 nM versus 50 nM, 12.32 ± 2.07% versus 46.99 ± 12.95%, two-tailed *t*-test, ****P = 3.577 × 10^−4^, *n* = 5) (Fig. 4b(i)). Without demultiplexing, it was found that there was a linear increase in percentage delayed events between 0.1 nM and 10 nM (*R*^2^ ≥ 0.988), followed by a plateau (Fig. 4b(ii)). The signal was demultiplexed using our alignment protocol, identifying individual populations of each barcoded probe. We then constructed bespoke concentration–percentage delay curves for each probe (Fig. 4b(iii)), and data points for each curve were normalized with background subtraction. We next tested our ability to quantify multiple miRNAs using a single-blinded test. A total of 40 barcoded probes were incubated with a mix of 40 miRNAs at various concentrations, ranging from 0.25 nM to 20 nM (Fig. 4c). The event files were sent to a blinded researcher who determined the percentage delay of each barcoded probe and interpolated the value with the standard curves to determine the concentration of the miRNAs. After estimating each miRNA concentration, the experiment was unblinded and compared to the actual concentration (Fig. 4c(i) and Supplementary Fig. 6). Comparison of each miRNA prediction to its true value can be made by comparing the colour changes vertically. Similar colours mean a close prediction of miRNA concentration to the true value. The ratio of predicted concentration:actual concentration was plotted, allowing comparison across all 40 barcoded probes; values >1 were overpredictions and <1 were underpredicted (Fig. 4c(ii)). The accuracy of all predictions was within one order of magnitude of the true concentration.

**Fig. 5:**
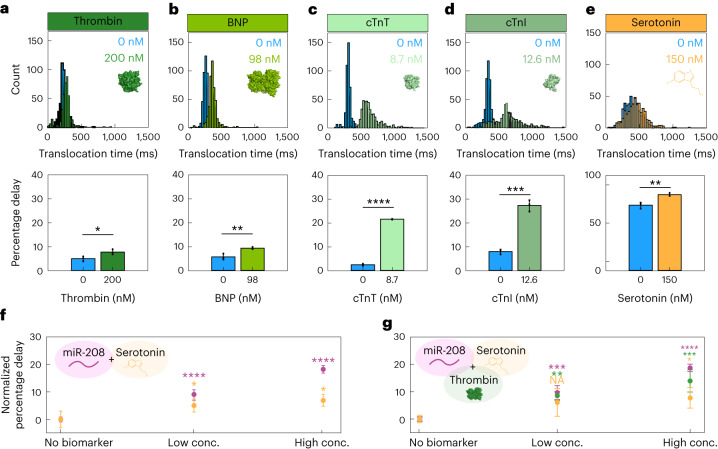
Protein and small molecule detection. **a**–**e**, Translocation time (top) and percentage delayed events (bottom) of barcoded probes (30 nM) ± each corresponding protein or small molecule: thrombin (*n* = 3, *n*_total events_ = 417 (0 nM), 1,341 (200 nM); *P = 0.0303) (**a**); BNP (*n* = 3, *n*_total events_ = 5,256 (0 nM), 1,040 (98 nM); **P = 0.0082) (**b**); cTnT (*n* = 3, *n*_total events_ = 8,920 (0 nM), 3,395 (8.7 nM); ****P = 1.62 × 10^−7^) (**c**); cTnI (*n* = 3, *n*_total events_ = 1,024 (0 nM), 1,024 (12.6 nM)); ***P = 1.99 × 10^−4^) (**d**); serotonin (*n* = 3, *n*_total events_ = 2,329 (0 nM), 1,602 (150 nM)); **P = 0.005) (**e**). Significance was determined by two-tailed *t*-tests. **f**, Multiplexed detection of serotonin and miR-208-5p. Low concentrations (conc.) (10 nM miR-208-5p, 150 nM serotonin) and high concentrations (50 nM miR-208-5p, 750 nM serotonin) were compared to the normalized control (no biomarker present) (ANOVA, *F* (2,9) = [96.04], ****P = 8.49 × 10^−7^, *F* (2,9) = [6.36], *P = 1.90 × 10^−2^, *n* = 3, *n*_total events_ = 65,545). **g**, Multiplexed detection of miR-208-5p, serotonin and thrombin. Low concentrations (10 nM miR-208-5p, 150 nM serotonin, 300 nM thrombin) and high concentration (50 nM miR-208-5p, 750 nM serotonin, 1,500 nM thrombin) were compared to the normalized control (no biomarker present). Normalized percentage delay of barcoded probes is shown (ANOVA, *F* (2,8) = [136.97], ****P = 6.48 × 10^−71^, *F* (2,8) = [4.74], *P = 4.38 × 10^−2^, *F* (2,8) = [28.57], ***P = 2.00 × 10^−4^, *n* = 3, *n*_total events_ = 44,956). All data are presented as mean ± s.d. All experiments were performed in sequencing buffer. NA, not applicable. Source data

This platform, due to its adaptability, has the potential to detect proteins, miRNAs and small molecules in a single experiment. Barcoded probes were designed with aptamers specific for each protein and small molecule (Supplementary Table 3; aptamer sequences were taken from published literature). In single barcoded probe experiments, a significant increase in translocation time could be observed when in the presence of its corresponding protein in pathophysiological concentrations: thrombin (Fig. 5a, *P = 0.030); B-type natriuretic peptide (Fig. 5b, **P = 0.008); cardiac troponin T (Fig. 5c, ****P = 1.62 × 10^−7^); and cardiac troponin I (Fig. 5d, ****P = 1.99 × 10^−4^, all two-tailed *t*-test). This corresponded with a significant increase in the percentage delayed events (Fig. 5a–d), suggesting that the detection of proteins is possible with this method. Moreover, a significant increase in translocation time and percentage delayed events was observed when serotonin (150 nM) was incubated with its corresponding barcoded probe (Fig. 5e, two-tailed *t*-test, **P = 0.005), despite our delay algorithm identifying a high number of delayed events in the control condition.

Multiplexed experiments were conducted by observing analytes of different molecular species. First, a duplex experiment of a small molecule and a miRNA was conducted. Incubation of barcoded probes with 50 nM small molecule and ≥3 nM miRNA significantly increased the percentage delayed events for each probe (Fig. 5f). We also performed a triplex experiment observing a miRNA, small molecule and protein simultaneously. The percentage delayed events for each barcoded probe increased significantly from the controls when miRNA (≥10 nM), small molecule (≥150 nM) and protein (≥50 nM) were present (Fig. 5g).

## Detection of miRNAs in human serum

**Fig. 6:**
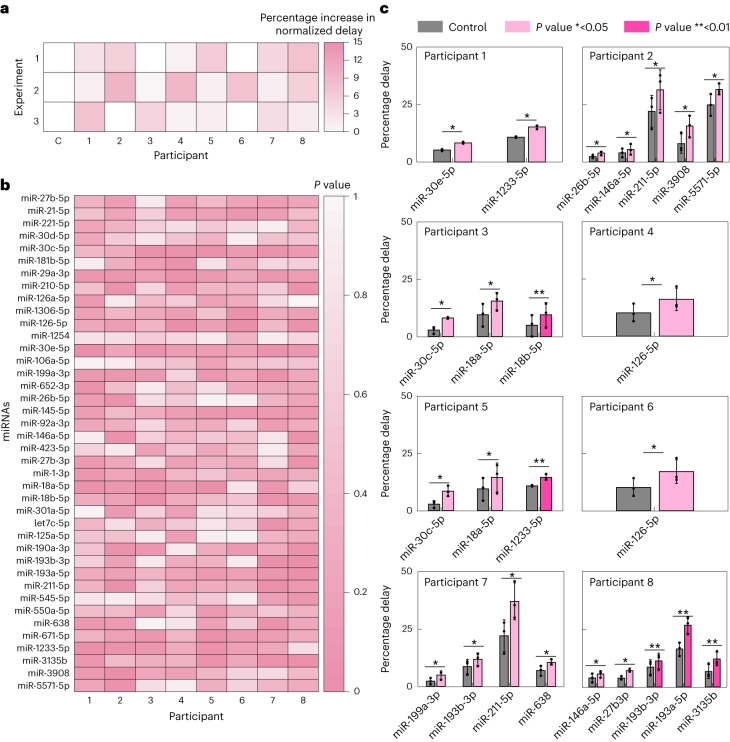
Multiplexed detection of miRNAs in human serum. **a**, Mean percentage delay of barcoded probes (not demultiplexed). Serum, after centrifugation with 10 kDa molecular weight cut-off spin filter, was incubated with 40 barcoded probes (30 nM) and compared to zero serum control (one-tailed *t*-test *P* > 0.05, *n* = 3, *n*_total events_ = 194,115). **b**, Heat map of *P* values comparing percentage delayed events ±serum (one-tailed *t*-test, *n* = 3, *n*_total events_ = 194,115). **c**, Significantly upregulated miRNAs per patient (one-tailed *t*-test *P ≤ 0.05, **P ≤ 0.01, *n* = 3, *n*_total events_ = 194,115). Exact *P* values are available from the source data file for this figure. Data are presented as mean ± s.d. All experiments were performed in sequencing buffer. Source data

Blood serum from eight healthy participants was tested for the presence of 40 miRNAs and compared to the negative control (no serum, only buffer). To reduce pore blockage (Supplementary Fig. 7), serum was centrifuged through a 10 kDa molecular weight cut-off spin filter to remove large proteins. The list of miRNAs selected for detection in this assay have all been previously associated with cardiovascular disease (Supplementary Table 1). The mean percentage delay of all miRNAs (not demultiplexed) was increased in each of the eight participant samples in three independent experiments; however, no significance was determined (Fig. 6a). Interestingly, when the signal was demultiplexed, significant changes in percentage delay of miRNAs could be observed (Fig. 6b). Of the 40 miRNAs tested, we saw a significant increase in 24 miRNAs across all eight participants (Fig. 6b,c and Supplementary Fig. 8). The most frequently observed miRNAs across all samples were: miR-1233-5p, miR146a-5p, miR211-5p, miR30c-5p, miR18a-5p, miR-126-5p and miR193b-3p. To further verify our results, we compared percentage delayed events with RT–qPCR of miR-29a in four samples. We found that our nanopore-based platform agreed with the RT–qPCR assay (Supplementary Fig. 9).

## Conclusions

There is a genuine clinical need to develop platforms that can rapidly detect multiple biomarkers in patient samples. By expanding the panel of molecules observed, it is possible that disease subpopulations could be identified and treatment regimens optimized. Detection of biomarkers of different molecular species within the same sample could also massively reduce the analysis pipeline.

Our data show that barcoded probes, in combination with the ONT sequencing platform, offer a highly accurate and sensitive analyte sensing platform with single-molecule resolution. We detected 40 different barcoded probes in the same sample and constructed independent concentration–delay curves. We then used these standard curves to predict miRNA concentrations in a single-blinded experiment. The multiplexed method was applied to multiple molecular species, allowing us to observe an increase in percentage delayed events due to proteins and small molecules (as well as miRNAs). Finally, we showed that the platform is compatible with human serum, indicating its potential as a biomarker detection platform.

The assay time can be less than 60 minutes in the platform’s current configuration. This is a clear improvement compared to other technologies, which take several hours or even days to complete^49^. ONT sequencing software can now spot specific barcode sequences, allowing for live demultiplexing, which will further reduce assay time and provide a critical advantage in the detection of analytes with short half-life. Moreover, since the MinION device is highly portable, assays could be performed away from the clinic. The speed and portability of the platform offer further advantages over other biomarker detection strategies.

The platform can correctly distinguish between barcodes with a single nucleotide change in the sequence. Interaction of probes was predicted (Supplementary Fig. 3); however, there was no clear relationship between dimer formation and event capture rate. This indicates that any future applications of this technology will not be limited by barcode sequences, but perhaps by the selectivity of the binding regions. Our data suggest that miRNA sequences with a similarity ≥90% have the potential to hybridize with the incorrect probe (Supplementary Fig. 10); strategies to distinguish between such miRNAs must therefore be further developed. There is also potential for the large number of biomarkers assessed to result in ‘data blindness’, to address this a graphical interface or risk-scoring method must be developed to assist the end user.

Currently, the detection limit of the platform for miRNA is approximately 50 pM. There is potential to push the technology further by reducing the false-negative rate of event alignment. At the moment, there are a large number of events that are rejected due to the thresholds applied to ensure high accuracy. One way to reduce this event loss and increase events available for delay analysis would be to switch to a different nanopore model.

A particular issue of assaying blood serum is that nanopore lumen are small and are easily blocked by proteins. Pore blockage reduces the barcoded probe capture rate, meaning assay times must be increased or repeated. To reduce the blockage of nanopores by proteins in this study, we added a filtration step, which removed the largest serum proteins (>10 kDa) but prevented simultaneous miRNA and protein detection in serum. In future applications, it would be preferable to develop our methodology further to reduce the sample processing required before detection whilst also reducing the unwanted pore blocking. Limiting proteolysis whilst preventing miRNA degradation is a key hurdle to overcome in the development of an assay that can detect both proteins and miRNAs in serum simultaneously.

The platform shows great potential for use in clinical environments, for example, to offer expansive, longitudinal disease tracking or early disease detection. The assay could be adapted for use with a variety of complex fluids, for example, saliva, urine and cerebrospinal fluid. With further optimization, this strategy could significantly reduce testing time, assay cost and sample volume, whilst increasing the data available to the clinician.

## Methods

### Chemicals and materials

All flow cells (MinION and Flongle) were provided by ONT. All probe sequences were custom designed. Barcoded probes and miRNAs were synthesized by Integrated DNA Technologies (Supplementary Tables 1 and 3). Proteins were obtained from the following: thrombin (Invitrogen, catalogue no. RP-43100); B-type natriuretic peptide (Bachem, catalogue no. 4095916); cardiac troponin I (Genscript Biotech, catalogue no. Z03320); cardiac troponin T (Ray Biotech, catalogue no. 230-00048). Small molecules: serotonin (Sigma-Aldrich, catalogue no. H9523). Ampure XP magnetic beads (for purification of DNA-barcoded probes) were purchased from Beckman Coulter. TA ligase was acquired from New England Biolabs. All chemicals used for buffer preparation were obtained from Sigma-Aldrich, Roche or VWR Chemicals. In all experiments, DNA lo-bind tubes were used (Eppendorf).

### Human donor blood serum

Human samples used in this research project were obtained from the Imperial College Healthcare Tissue Bank (ICHTB). ICHTB is supported by the National Institute for Health Research (NIHR) Biomedical Research Centre based at Imperial College Healthcare NHS Trust and Imperial College London. ICHTB is approved by Wales REC3 to release human material for research (22/WA/0214), and the samples for this project (R22016) were issued from subcollection reference number NHL_FN_021_028.

Venous blood was collected in red-topped vacutainers (Beckton Dickinson) and allowed to clot at room temperature for 15 min before centrifugation at 3,000*g*, 15 min, 4 °C. The resulting serum was then aliquoted into small volumes and frozen at −80 °C until use. The serum was filtered using a 10 kDa molecular weight cut-off spin filter (Sartorius) before incubation with barcoded probes.

### Barcoded probe design

The complete carrier design consists of three sections named ‘adapter’, ‘barcode region’ and ‘target binding region’ (Fig. 1a); when fully assembled, this was called a ‘barcoded probe’. The adapter section is identical for all probes. It is the ONT Y-adapter, which consists of (1) a leader (which facilitates threading into the nanopore); (2) a tether (to enhance the capture rate); and (3) a motor protein (to control the translocation of the barcoded probe through a nanopore). The adapter section is ligated to the 5′ end of all barcoded probes, ensuring that translocation events are always in the 5′ to 3′ direction. The barcode region consists of a polynucleotide identifier and is followed by spacer nucleotides (to separate the barcode and target binding regions). The target binding region consists of a DNA aptamer or complementary miRNA sequence, depending on the species of the target analyte.

### Barcoded probe preparation

Each probe was incubated with ligation complementary strand in a molar ratio of 1:3 in nuclease-free water at room temperature for 1 h. The resultant mix was combined with 10 nM adapter and an equal volume of TA ligase master mix (New England Biolabs). The mix was centrifuged at 4 °C for 1 min and then incubated at room temperature for 20 min. Probes were purified using the solid phase reversible immobilization method. Ampure XP beads (Beckman Coulter) were added at 1.4 times the total solution volume. The beads (with probes bound) were washed two times with a short fragment buffer (ONT). After the washes, the beads were resuspended in nuclease-free water, causing the probes to be released. A 100 nM tether was then added to the probes along with sequencing buffer. The 2× sequencing buffer contained 700 mM KCl, 50 mM HEPES, 100 mM MgCl_2_, 100 mM ATP, 4.4 mM EDTA (pH 8.0). Barcoded probes were incubated with the target analyte for 30 min at room temperature before loading into flow cells. The concentration of the barcoded probes in all experiments was 30 nM, as it was more sensitive than 100 nM (Supplementary Fig. 11).

Preprepared mixes of barcoded probes are resilient to multiple freeze–thaw cycles and long-term storage at −20 °C (Supplementary Fig. 12). Hybridization dynamics of miRNAs with barcoded probes were also investigated, revealing that incubation of 5–10 minutes is sufficient to reach equilibrium for probe–analyte binding (Supplementary Fig. 13). This can potentially reduce the sample preparation time and assay variability significantly. A gel binding assay was performed to confirm interactions of barcoded probes and their corresponding miRNAs (Supplementary Fig. 14).

### Sequencing experiments and data acquisition

All sequencing experiments were performed at 34 °C using either the MinION or Flongle sequencing device (ONT). The MinION/Flongle was connected through a USB 3.0 port to a PC with a minimum of 16 GB RAM. A membrane check was performed before each run to determine the integrity of the membrane and to identify how many nanopores were active. Before each experiment, flow cells were flushed with 2× 500 µl (MinION) or 1× 150 µl (Flongle) sequencing buffer. The volume for each sequencing experiment was determined by the flow cell used: 150 µl (MinION) or 30 µl (Flongle). After each experiment, the MinION flow cell was washed with the flow cell wash kit (ONT) according to the manufacturer’s protocol.

Data collection was performed using the proprietary software MinKNOW (ONT). Base calling was either performed in real time (MinKNOW) or offline within a custom-written MATLAB script, ‘The Nanopore App’, previously published by our group^43^.

### Event analysis

Barcoded probe translocations were identified and analysed using the following workflow: (1) event identification; (2) event base calling; (3) event alignment; (4) event delay analysis.

Event identification included the tracking and then subtraction of the baseline signal. A cut-off threshold was then determined on the basis of the background noise (30–40 standard deviations above mean noise level). ‘Peakfinder’ function in MATLAB was used to spot events. Any events identified that were shorter than 100 ms were excluded at this stage.

Event base calling was performed with the Guppy base caller using the high accuracy flip-flop model (ONT).

Each base-called event was aligned against a library of barcode sequences. Each event was attributed to one of the library sequences on the basis of an alignment scoring method. We found that the accuracy of the Guppy base caller was much more accurate at the 5′ end of the barcode sequence. Further thresholds were applied to ensure that only true positive barcode events were retained for further analysis. These thresholds were: (1) sequence starts with ‘GGG’, (2) ≥15 bases aligned to the library sequence; (3) one mismatched base in the first ten bases; and (4) ≤5 mismatched bases in the entire sequence. Further information is in Supplementary Methods (‘Event selection pipeline’).

To distinguish between analyte-bound and unbound barcoded probe events, we performed analysis of each event. When an analyte is bound to the barcoded probe, there is a ‘quiet’ sojourn in the electrical signal, which persists until the analyte is dissociated from the probe, at which point it can complete its translocation. An event is defined as delayed if the moving standard deviation of the signal is less than the threshold of 0.003 for a period greater than 10 bins (each event signal is separated into a total of 75 bins). All other events are defined as having no delay.

### Statistical tests

Where possible, a Kolmogorov–Smirnov test was performed to determine whether our data were normally distributed at the 5% significance level.

One-sample *t*-tests, were performed with *n* − 1 degrees of freedom unless otherwise stated. Details are indicated in the figure legends. No statistical methods were used to predetermine sample sizes.

The experiments performed to generate the data in Fig. 4c were single-blinded (the analyst was blinded to sample contents). The experiments performed to generate the data in Fig. 6 and Supplementary Figs. 8 and 9 were fully blinded to all researchers.

### Reporting summary

Further information on research design is available in the Nature Portfolio Reporting Summary linked to this article.

## Online content

Any methods, additional references, Nature Portfolio reporting summaries, source data, extended data, supplementary information, acknowledgements, peer review information; details of author contributions and competing interests; and statements of data and code availability are available at 10.1038/s41565-023-01479-z.

### Supplementary information

Supplementary Information Supplementary Tables 1–3, Figs. 1–14 and Methods.

Reporting Summary

Supplementary Data 1 Source data for Supplementary Figs. 2–13.

### Source data

Source Data Fig. 2 Statistical source data.

Source Data Fig. 3 Statistical source data.

Source Data Fig. 4 Statistical source data.

Source Data Fig. 5 Statistical source data.

Source Data Fig. 6 Statistical source data.

## Data Availability

Source data are provided with this paper. Further data supporting the plots within this paper and other study findings are available in the supplementary files or by contacting the corresponding authors.

## Code availability

The Nanopore App used to analyse the data is available at https://www.imperial.ac.uk/people/joshua.edel or by request to the corresponding authors.
