# Supplementary material for: Nanopore sequencing of DNA-barcoded probes for highly multiplexed detection of microRNA, proteins and small biomarkers
Source: Nat Nanotechnol. 2023 Sep 25;18(12):1483–91. doi: 10.1038/s41565-023-01479-z (PMC10716039; doi:10.1038/s41565-023-01479-z)
Supplement: Supplementary file 1 — Supplementary Tables 1–3, Figs. 1–14 and Methods. [file 41565_2023_1479_MOESM1_ESM.pdf]

nature nanotechnology

Article https://doi.org/10.1038/s41565-023-01479-z

# Nanopore sequencing of DNA-barcoded probes for highly multiplexed detection of microRNA, proteins and small biomarkers

In the format provided by the authors and unedited

## Supplementary Data Table of Contents

Supplementary Data Table of Contents ........ 1
Supplementary Tables ........ 2
Supplementary Table 1| Table of barcoded probe sequences. ........ 2
Supplementary Table 2| Alignment of barcoded probe 38 sequenced events against library sequence. ........ 3
Supplementary Table 3| Protein and small molecule barcoded probes. ........ 4
Supplementary Figures ........ 5
Supplementary Fig. 1| Delay detection method using moving standard deviation. ........ 5
Supplementary Fig. 2| Statistical analysis of barcoded probe 38 events. ........ 6
Supplementary Fig. 3| Prediction of barcoded probe interaction. ........ 7
Supplementary Fig. 4| Sequence similarity of miRNAs in multiplexed experiments. ........ 8
Supplementary Fig. 5| Individual barcoded probe concentration- delay relationships. ........ 9
Supplementary Fig. 6| Individual barcoded probe concentration- delay relationships. ........ 10
Supplementary Fig. 7| Effect of serum on pore blockage. ........ 11
Supplementary Fig. 8| Analysis of patient samples. ........ 12
Supplementary Fig. 9| RT-qPCR compared to nanopore experiments. ........ 13
Supplementary Fig. 10| Probe selectivity of miRNAs. ........ 14
Supplementary Fig. 11| Effect of barcoded probe concentration on assay sensitivity. ........ 15
Supplementary Fig. 12| Lifetime analysis of frozen barcoded probes. ........ 16
Supplementary Fig. 13| Barcoded probe binding to miRNAs equilibrates rapidly. ........ 16
Supplementary Fig. 14| Barcoded probe binding to miRNAs- gel assay. ........ 18
Supplementary methods ........ 19
Event selection pipeline ........ 19
Uncropped gel images ........ 20
Manual Nanopore App ........ 22

## Supplementary Tables

### Supplementary Table 1| Table of barcoded probe sequences.

All probes were purchased from IDT with high purity and desalting. iSpC3= C3 Spacer phosphoramidite. Associated cardiac diseases: ACS Acute Coronary syndrome; AMI Acute Myocardial Infarction; AS Aortic Stenosis; ASVD Atherosclerosis; CAD Coronary Artery Disease; CF Cardiac Fibrosis; DCM Dilated cardiomyopathy; HF Heart failure; HFpEF Heart failure with preserved ejection fraction; HFrEF Heart failure with reduced ejection fraction; Hyp Hypertension; NSTEMI Non-ST-elevated myocardial infarction; SCD Sudden cardiac death; STEMI ST-elevated myocardial infarction. $K_d$ and $V_{max}$ values were obtained by fitting multiplexed experiments with the the Hill fit with $n_H = 1$.

5′-Target binding region-3′ 5′-DNA barcode sequencing region-3′

| Number | Barcode name | Target sequence (miRNA sequence) | Associated cardiac disease | Binding sequence | Barcode sequence | Multiplexed $K_d$ (nM) | Multiplexed $V_{max}$ |
|---|---|---|---|---|---|---|---|
| 1 | Adapter_barcode_c-hsa-miR-27b-5p | CAAGTGGTTAGTCGATTCGAGA | CF / Hyp / ACS / HF | GTTCACCAATCAGCTAAGCTCT | GGGATCGCTACGCCTTCGGCTCGTAATCATAGTCGAGT | 0.976 | 48.445 |
| 2 | Adapter_barcode_c-hsa-miR-21-5p | AGTTGTAGTCAGACTATTCGAT | NSTEMI / ASVD | TCAACATCAGTCTGATAAGCTA | GGGAGCTCAGAGCAGGTCACTCAAGATACGAGCTGCGT | 4.172 | 62.005 |
| 3 | Adapter_barcode_c-hsa-miR-221-5p | TTTAGATGTAACATACGGTCCA | SCD | AAATCTACATTGTATGCCAGGT | GGGGTAAGTCTGCATCAGCGCGCGGCTGTGCGAGGATA | 2.107 | 32.552 |
| 4 | Adapter_barcode_c-hsa-miR-30d-5p | GAAGGTCAGCCCCTACAAATGT | STEMI | CTTCCAGTCGGGGATGTTTACA | GGGCTACGACAGTACGCTAGCAAGGATAGACACTACGA | 9.060 | 64.651 |
| 5 | Adapter_barcode_c-hsa-miR-30c-5p | CGACTCTCACATCCTACAAATGT | CF / HF | GCTGAGAGTGTAGGATGTTTACA | GGGTACTGAACACAAGTTCGTCGTCGAGCAATCACAAT | 14.363 | 34.463 |
| 6 | Adapter_barcode_c-hsa-miR-181b-5p | TGGGTGGCTGTCGTTACTTACAA | AS | ACCCACCGACAGCAATGAATGTT | GGGCTAGTGCGCAGTTGTCTCGGCGGAGTTGAGACTGA | 1.453 | 62.109 |
| 7 | Adapter_barcode_c-hsa-miR-29a-3p | ATTGGCTAAAGTCTACCACGAT | CF / HF/ STEMI | TAACCGATTTCAGATGGTGCTA | GGGGATCATGGTAGTCTTCAAGATCGAGTATGTCTGTC | 1.881 | 24.849 |
| 8 | Adapter_barcode_c-hsa-miR-210-5p | GTCACACGCCACCCGTCCCCGA | HF | CAGTGTGCGGTGGGCAGGGGCT | GGGGTTCACATCAAGGTCATACCGCGAGTTCTATTTTA | 1.407 | 22.671 |
| 9 | Adapter_barcode_c-hsa-miR-126-5p | GCGCATGGTTTTCATTATTAC | HF | CGCGTACCAAAAGTAATAATG | GGGGCTTGGGGGATAGATGTGCCCCGCGCATCGGACCT | 0.832 | 47.446 |
| 10 | Adapter_barcode_c-hsa-mir-1306-5p | ACCTGCAAACGTCCCCTCCACC | AMI | TGGACGTTTGCAGGGGAGGTGG | GGGATGACACACGTTTTCGATAGGGACGCCGACTTTAA | 0.126 | 27.487 |
| 11 | Adapter_barcode_c-hsa-miR-126-5p | GCGCATGGTTTTCATTATTAC | HF | CGCGTACCAAAAGTAATAATG | GGGTGATAATAAGACCTGACAGACAATAGGGAGAACTC | 0.193 | 56.250 |
| 12 | Adapter_barcode_c-hsa-miR-1254 | TGACGTCCGAGGTCGAAGGTCCGA | HF | ACTGCAGGCTCCAGCTTCCAGGCT | GGGTTAGTAATCAAGTCTGATCGTAATAGCTAAGTCAT | 1.491 | 57.875 |
| 13 | Adapter_barcode_c-hsa-miR-30e-5p | GAAGGTCAGTTCCTACAAATGT | DCM / AS | CTTCCAGTCAAGGATGTTTACA | GGGCCTGTTCTAATTGCGCGGAGAGGCGAGATGTTTCT | 2.244 | 41.573 |
| 14 | Adapter_barcode_c-hsa-miR-106a-5p | GATGGACGTGACATTCGTGAAAA | DCM | CTACCTGCACTGTAAGCACTTTT | GGGTGGGATACTGACGTGCCGGAATACCCAGACGTGCC | 2.451 | 30.826 |
| 15 | Adapter_barcode_c-hsa-miR-199a-3p | ATTGGTTACACGTCTGATGACA | AMI / HF | TAACCAATGTGCAGACTACTGT | GGGTGATCGGCACCTAAACGCATTAGCCCTGCAATACG | 1.443 | 41.053 |
| 16 | Adapter_barcode_c-hsa-miR-652-3p | GTGTTGGGATCACCGCGGTAA | ACS | CACAACCCTAGTGGCGCCATT | GGGAGCTGCTCGGAAGCCATAAGGTACTTTAATTTGGG | 2.682 | 18.111 |
| 17 | Adapter_barcode_c-hsa-miR-26b-5p | TGGATAGGACTTAATGAACTT | AS | ACCTATCCTGAATTACTTGAA | GGGCACGGATTTCTATATTGCTCAACCAGGCAGCGCAA | 0.216 | 49.819 |
| 18 | Adapter_barcode_c-hsa-miR-145-5p | TCCCTAAGGACCCTTTTGACCTG | ACS / CAD / STEMI | AGGGATTCCTGGGAAAACTGGAC | GGGATTGTGCGCTTTTCTCCATGCGTCTTAGACATCTC | 5.260 | 64.427 |
| 19 | Adapter_barcode_c-hsa-miR-92a-3p | TGTCCGGCCCTGTTCACGTTAT | ACS | ACAGGCCGGGACAAGTGCAATA | GGGGTGAAATCCCCGTCTAGGTTATGGCTGGGGGGATT | 0.866 | 30.393 |
| 20 | Adapter_barcode_c-hsa-miR-146a-5p | TTGGGTACCTTAAGTCAAGAGT | ACS / AMI / STEMI / HF | AACCCATGGAATTCAGTTCTCA | GGGCGTAAACTTATCACGACACAATGAACAAGCCTGCA | 0.568 | 60.163 |
| 21 | Adapter_barcode_c-hsa-miR-423-5p | TTTCAGAGCGAGAGACGGGGAGT | HF | AAAGTCTCGCTCTCTGCCCCTCA | GGGGGCAGTGTCCGAGCGTCCTCAATCATGAGCGATTC | 2.041 | 38.207 |
| 22 | Adapter_barcode_c-hsa-miR-27b-3p | CGTCTTGAATCGGTGACACTT | AS | GCAGAACTTAGCCACTGTGAA | GGGCGTCCAGACTTAATGTCTGCTCACTGACATCGCGA | 2.468 | 52.598 |
| 23 | Adapter_barcode_c-hsa-miR-1-3p | TATGTATGAAGAAATGTAAGGT | NSTEMI /ACS | ATACATACTTCTTTACATTCCA | GGGATTAGCGGAACCAAACCCAGGAAGGCTTGAAGGCG | 0.626 | 14.264 |
| 24 | Adapter_barcode_c-hsa-miR-18a-5p | GATAGACGTGATCTACGTGGAAT | CAD | CTATCTGCACTAGATGCACCTTA | GGGAACCTTAGGGGCCTCGAATCTTTGAGACGACTAGG | 0.587 | 6.944 |
| 25 | Adapter_barcode_c-hsa-miR-18b-5p | GATTGACGTGATCTACGTGGAAT | HF | CTAACTGCACTAGATGCACCTTA | GGGTAATTACTGCCCCACCATGACATTTTAATAGCAGT | 0.992 | 30.556 |
| 26 | Adapter_barcode_c-hsa-miR-301a-5p | TCATCACGTTATTTCAGTCTCG | HF | AGTAGTGCAATAAAGTCAGAGC | GGGGACCTTGAGACAGAACTTATCAATGTACAACTGAA | 1.081 | 30.850 |
| 27 | Adapter_barcode_c-hsa-let7c-5p | TTGGTATGTTGGATGATGGAGT | CAD | AACCATACAACCTACTACCTCA | GGGCGAAGGATTGGCCCCCCGATTACCACCGCCGTGAG | 0.306 | 39.098 |
| 28 | Adapter_barcode_c-hsa-miR-125a-5p | AGTGTCCAATTTCCCAGAGTCCCT | HFrEF | TCACAGGTTAAAGGGTCTCAGGGA | GGGAATTGCCAACAGGTCAAGCCCTGTTCTCACTGGTC | 0.251 | 52.617 |
| 29 | Adapter_barcode_c-hsa-miR-190a-3p | TCCTTATACAAACTATATATC | HFpEF | AGGAATATGTTTGATATATAG | GGGCAGTGCTTGCCCCCAGTAGAGTGTGGAAGGGCATA | 0.692 | 24.663 |
| 30 | Adapter_barcode_c-hsa-miR-193b-3p | TCGCCCTGAAACTCCCGGTCAA | HFrEF /STEMI | AGCGGGACTTTGAGGGCCAGTT | GGGAAAGCCCACTCTCCACACTTCAAGGTTAAATGGCG | 6.454 | 21.231 |
| 31 | Adapter_barcode_c-hsa-miR-193a-5p | AGTAGAGCGGGCGTTTCTGGGT | HF | TCATCTCGCCCGCAAAGACCCA | GGGGACTAATACAATCGGAAGCAACTCTCACGCCGCAC | 2.872 | 50.154 |
| 32 | Adapter_barcode_c-hsa-miR-211-5p | TCCGCTTCCTACTGTTTCCCTT | HFrEF / ASVD | AGGCGAAGGATGACAAAGGGAA | GGGCTACGATTCATGTCTCCCCCCACATATGATTGATC | 0.755 | 40.044 |
| 33 | Adapter_barcode_c-hsa-miR-545-5p | AGTAGATTATTTGTAAATGACT | HFpEF / STEMI | TCATCTAATAAACATTTACTGA | GGGTCGGGCGCTTAATCGCAATGTTCATCCGGAACGGA | 0.704 | 57.095 |
| 34 | Adapter_barcode_c-hsa-miR-550a-5p | CCCGAGAATGAGGGAGTCCGTGA | HFpEF / HF | GGGCTCTTACTCCCTCAGGCACT | GGGTAAATTCCACATTACGATAGCTGACGTCCTGGTAG | 4.246 | 8.531 |
| 35 | Adapter_barcode_c-hsa-miR-638 | TCCGGCGGTGGGCGGGCGCTAGGGA | HFrEF / HFpEF | AGGCCGCCACCCGCCCGCGATCCCT | GGGCACCCCCTAGCCTGACCTTAAATGATAATTCCTGT | 1.056 | 45.593 |
| 36 | Adapter_barcode_c-hsa-miR-671-5p | GAGGTCGGGGAGGTCCCGAAGGA | HFrEF | CTCCAGCCCCTCCAGGGCTTCCT | GGGTTTAGCCTAACCTCTGATGCATTGCACCGGAGTTC | 21.486 | 69.518 |
| 37 | Adapter_barcode_c-hsa-miR-1233-5p | ACGGCACGGGACCGGAGGGTGA | HFpEF | TGCCGTGCCCTGGCCTCCCACT | GGGCCCAATATACGCTGAACCTTCCATCCGATTTTCAG | 0.297 | 52.885 |
| 38 | Adapter_barcode_c-hsa-miR-3135b | GTGGTGACGTGAGCGAGGTCGG | HF | CACCACTGCACTCGCTCCAGCC | GGGCCTTGGAATTAGAACCGTGTGATTCTACGCCTAGG | 1.774 | 61.030 |
| 39 | Adapter_barcode_c-hsa-miR-3908 | TTTGTCAGATGGATGTAACGAG | HF | AAACAGTCTACCTACATTGCTC | GGGAGGACTTCTTCGGTGTAATCGGAGTACATCAATGT | 0.767 | 44.722 |
| 40 | Adapter_barcode_c-hsa-miR-5571-5p | CCCTCCGAGGAAACTCTTAAC | HF | GGGAGGCTCCTTTGAGAATTG | GGGAACGTACTTTGTGGGGATAAGCTGTACAGGGCTGT | 1.634 | 41.139 |

**Supplementary Table 2| Alignment of barcoded probe 38 sequenced events against library sequence.** The most common basecalled sequence, is shown in row two. The percentage value presents the occurrence of the most common base at that position in the alignment (n=30). The critical alignment region (bases n = 15) was used to classify barcodes. Bases with an alignment score of <90% were removed from the table, as they were irrelevant for barcode classification.

Critical alignment region

DNA barcode sequencing region (5' – 3')

| | | | | | | | | | | | | | | | | | | | | | | | | | | |
|---|---|---|---|---|---|---|---|---|---|---|---|---|---|---|---|---|---|---|---|---|---|---|---|---|---|---|
| Library sequence Barcode 38 | G | G | G | C | C | T | T | G | G | A | A | T | T | A | G | A | A | C | C | G | T | G | T | G | A | T | T |
| Most common sequence | G | G | G | C | C | T | T | G | G | A | A | T | T | A | G | A | A | C | C | G | T | G | T | G | A | T | T |
| % | 100 | 100 | 100 | 100 | 100 | 99 | 100 | 99 | 99 | 98 | 99 | 98 | 99 | 95 | 93 | 95 | 97 | 96 | 96 | 91 | 91 | 90 | 91 | 90 | 90 | 91 | 93 |
| Sequence event 1 | G | G | G | C | C | T | T | G | G | A | A | T | T | A | G | A | A | C | C | G | T | G | T | G | A | T | T |
| Sequence event 2 | G | G | G | C | C | T | T | G | G | A | A | T | T | A | G | A | A | C | C | G | T | G | T | G | A | T | T |
| Sequence event 3 | G | G | G | C | C | T | T | G | G | A | A | T | T | A | G | A | A | C | C | G | T | G | T | G | A | T | T |
| Sequence event 4 | G | G | G | C | C | T | T | G | G | A | A | T | T | A | G | A | A | C | C | G | T | G | T | G | A | T | T |
| Sequence event 5 | G | G | G | C | C | T | T | G | G | A | A | T | T | A | G | A | A | C | C | G | T | G | T | G | A | T | T |
| Sequence event 6 | G | G | G | C | C | T | T | G | G | A | A | T | T | A | G | A | A | C | C | G | T | G | T | G | A | T | T |
| Sequence event 7 | G | G | G | C | C | T | T | G | G | A | A | T | T | A | G | A | A | C | C | G | T | G | T | G | A | T | T |
| Sequence event 8 | G | G | G | C | C | T | T | G | G | A | A | T | T | A | G | A | A | C | C | G | T | G | T | G | A | T | T |
| Sequence event 9 | G | G | G | C | C | T | T | G | G | A | A | T | T | A | G | A | A | C | C | G | T | G | T | G | A | T | T |
| Sequence event 10 | G | G | G | C | C | T | T | G | G | A | A | T | T | A | G | A | A | C | C | G | T | G | T | G | A | T | T |
| Sequence event 11 | G | G | G | C | C | T | T | G | G | A | A | T | T | A | A | G | A | A | C | C | G | T | G | T | G | A | T |
| Sequence event 12 | G | G | G | C | C | T | T | G | G | A | A | T | T | A | G | A | A | C | C | G | T | G | T | G | A | T | T |
| Sequence event 13 | G | G | G | C | C | T | T | G | G | A | A | T | T | A | G | A | C | C | G | T | G | T | G | A | T | T | C |
| Sequence event 14 | G | G | G | C | C | T | T | G | G | A | A | T | T | A | G | A | A | C | C | G | T | G | T | G | A | T | T |
| Sequence event 15 | G | G | G | C | C | T | T | G | G | A | A | T | T | A | G | A | A | C | C | G | T | G | T | G | A | T | T |
| Sequence event 16 | G | G | G | C | C | T | T | G | G | A | A | T | T | G | A | T | T | C | C | A | A | C | C | T | A | G | A |
| Sequence event 17 | G | G | G | C | C | T | T | G | G | A | A | T | T | A | G | A | A | C | C | G | T | G | T | G | A | T | T |
| Sequence event 18 | G | G | G | C | C | T | T | G | G | A | A | T | T | A | G | A | A | C | C | G | T | G | T | G | A | T | T |
| Sequence event 19 | G | G | G | C | C | T | T | G | G | A | A | T | T | A | G | A | A | C | C | G | T | G | T | G | A | T | T |
| Sequence event 20 | G | G | G | C | C | T | T | G | G | A | A | T | T | A | G | A | A | C | C | G | T | G | T | G | A | T | T |
| Sequence event 21 | G | G | G | C | C | T | T | G | G | A | A | T | T | A | G | A | A | C | C | G | T | G | T | G | A | T | T |
| Sequence event 22 | G | G | G | C | C | T | T | G | G | A | A | T | T | A | G | A | A | C | C | G | T | G | T | G | A | T | T |
| Sequence event 23 | G | G | G | C | C | T | T | G | G | A | A | T | T | A | A | G | A | C | A | A | G | T | T | C | T | G | T |
| Sequence event 24 | G | G | G | C | C | T | T | G | G | A | A | T | T | A | G | A | A | C | C | G | T | G | T | G | A | T | T |
| Sequence event 25 | G | G | G | C | C | T | T | G | G | A | A | T | T | A | G | A | A | C | C | G | T | G | T | G | A | T | T |
| Sequence event 26 | G | G | G | C | C | T | T | G | G | A | A | T | T | A | G | G | A | C | C | G | T | G | T | G | A | T | T |
| Sequence event 27 | G | G | G | C | C | T | T | G | G | A | A | T | T | A | G | A | A | C | C | C | G | T | G | T | G | A | T |
| Sequence event 28 | G | G | G | C | C | T | T | G | G | A | A | T | T | A | G | A | A | C | C | G | T | G | T | G | A | T | T |
| Sequence event 29 | G | G | G | C | C | T | T | G | G | A | A | T | T | A | G | A | A | C | C | G | T | G | T | G | A | G | T |
| Sequence event 30 | G | G | G | C | C | C | T | T | G | G | A | A | T | T | A | G | A | A | C | C | G | T | G | T | G | A | T |

**Supplementary Table 3| Protein and small molecule barcoded probes.**

All probes were purchased from IDT with high purity and desalting.

| | 5′ - DNA barcode sequencing region - 3′ | 5′ - Target binding region - 3′ | |
|---|---|---|---|
| **Barcode name** | **Barcode sequence** | **Aptamer sequence** | **Aptamer reference** |
| Adapter_barcode_Thrombin | GGGTTAAGCTATTGCTAACTGTAGTCCTAGTCTAGCTA | AGTCCGTGGTAGGGCAGGTTGGGGTGACT | Müller et al. (2008) J. Thromb. Haemost. |
| Adapter_barcode_BNP | GGGCGTAAACGTGAATGGGAGCCAATCATATACGTTGG | ATAACGACATCCGCCGGCACGAAGGGATCAAGTCGATAGG | Wang et al. (2014) Microchimica. Acta. |
| Adapter_barcode_cTnT | GGGCTTAAGTTCGGTCATTAACAGTGTCAATCTTGCCA | ATACGGGAGCCAACACCAGGACTAACATTATAAGAATTGCGAATAATCATTGGAGAGCAGGTGTGACGGAT | Sharma and Jang (2019) Sci. Rep. |
| Adapter_barcode_cTnI | GGGATTCAGATCTTTGACCGATCGTACTGACGTGTACG | AGTCTCCGCTGTCCTCCCGATGCACTTGACGTATGTCTCACTTTCTTTTCATTGACATGGGATGACGCCGTGACTG | Negahdary et al. (2017) Sens. Actuators B Chem. |
| Adapter_barcode_Serotonin | GGGACTAGATAAAAGGAAGGGAGCACAGTAACGTCGTT | CGACTGGTAGGCAGATAGGGGAAGCTGATTCGATGCGTGGGTCG | Nakatsuka et al. (2018) Science |

## Supplementary Figures

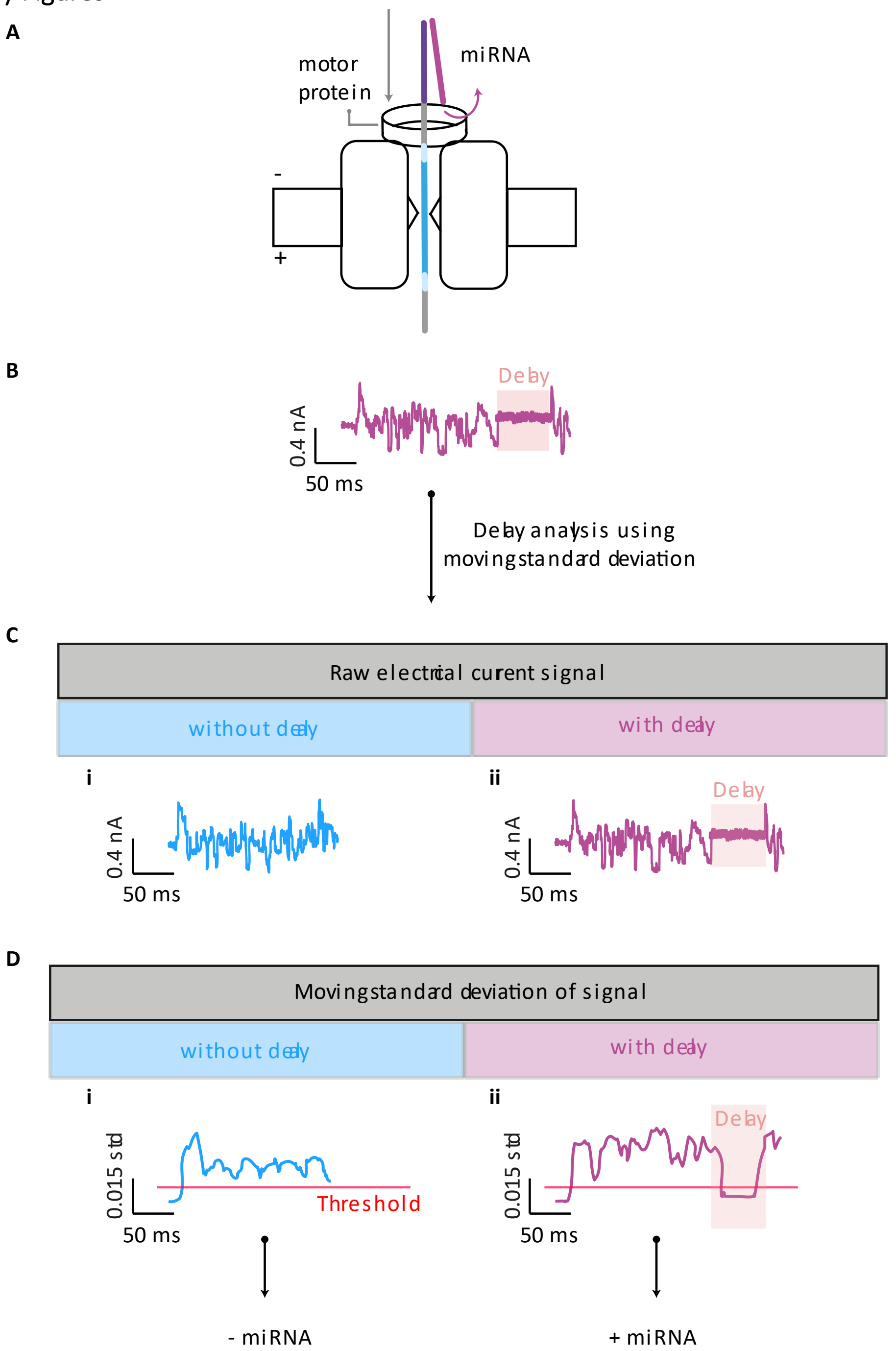


**Supplementary Fig. 1| Delay detection method using moving standard deviation.**
(A) Illustration of barcoded probe translocation through a single nanopore. miRNA is bound to a section of the barcoded probe containing the complementary miRNA sequence. Double-stranded DNA / RNA is too large to translocate through the nanopore. The motor protein unzips the double-strand to allow the single-stranded barcoded probe to complete its translocation. (B) The period of strand unzipping can be observed in the electrical current as a "delay". (C) Example electrical current signals of barcode translocations without (C,i) and with (C,ii) delay periods. (D) Moving standard deviation of signal traces in part C. When the moving standard deviation drops below a threshold of 0.003 for a period of ≥10 bins (out of 75 bins total), the event is classified as delayed. Example values for events with (D,i) and without (D,ii) delay are shown.

A

B

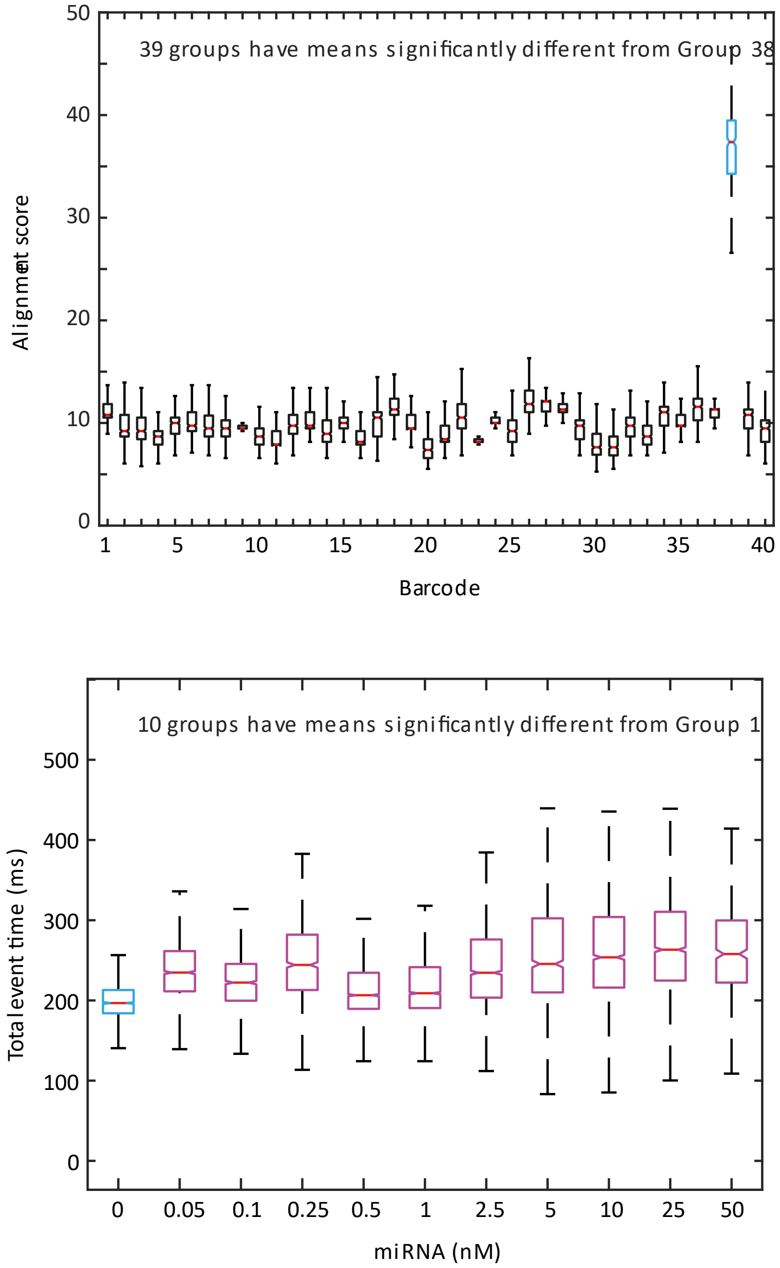


**Supplementary Fig. 2| Statistical analysis of barcoded probe 38 events.**

(A) Alignment score of barcoded probe 38 events when aligned against all 40 barcoded probe sequences. Alignment score for the barcoded probe 38 sequence was significantly higher from all other barcode sequences (ANOVA, F (39,17360) = [3441.41], ****p=0, N=3, $n_{total\ events}$ = 454). (B) Total event time (ms) of barcoded probe 38 events in the presence of various concentrations (0.05 to 50 nM) of its corresponding miRNA. Total event time was significantly increased in all conditions where miRNA was present when compared to control (ANOVA, F (10, 19691) = [240.65], ****p=0, N=5, $n_{total\ events}$= 54197 ). Summary statistics for boxplots (A) and (B): centre = median, bounds of box = interquartile range (IQR) 25$^{th}$ and 75$^{th}$ percentile, whiskers = minimum and maximum within range of 1.5 IQR, outliers outside of 1.5 IQR have been removed).

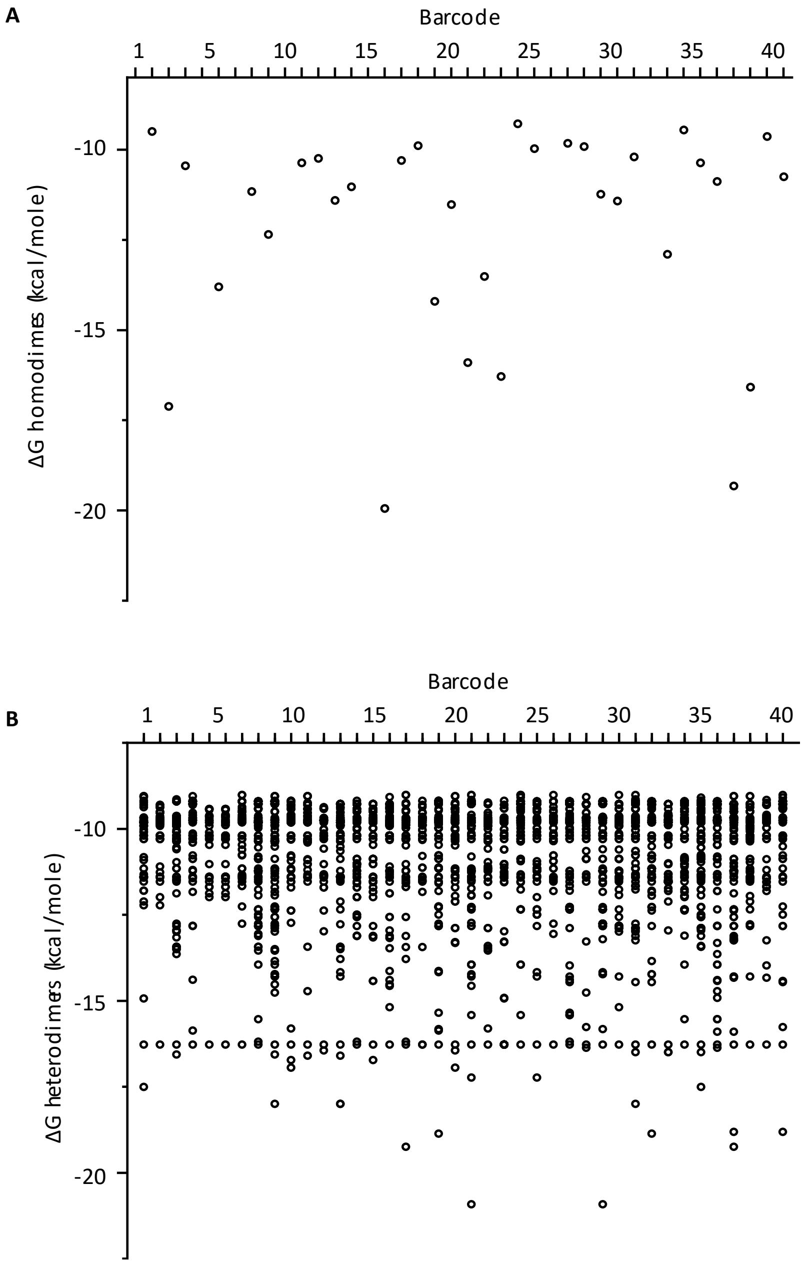


**Supplementary Fig. 3| Prediction of barcoded probe interaction.**

Theoretical barcoded probe interactions were determined using an online web-app (Integrated DNA Technologies OligoAnalyser$^{TM}$ tool). (A) Free energy of predicted homo-dimerisation of barcoded probes. Homo-dimers with ΔG ≤-9 kcal/ mol are shown. (B) Free energy of predicted hetero-dimerisation of barcoded probes. Hetero-dimers with ΔG ≤-9 kcal/mol are shown.

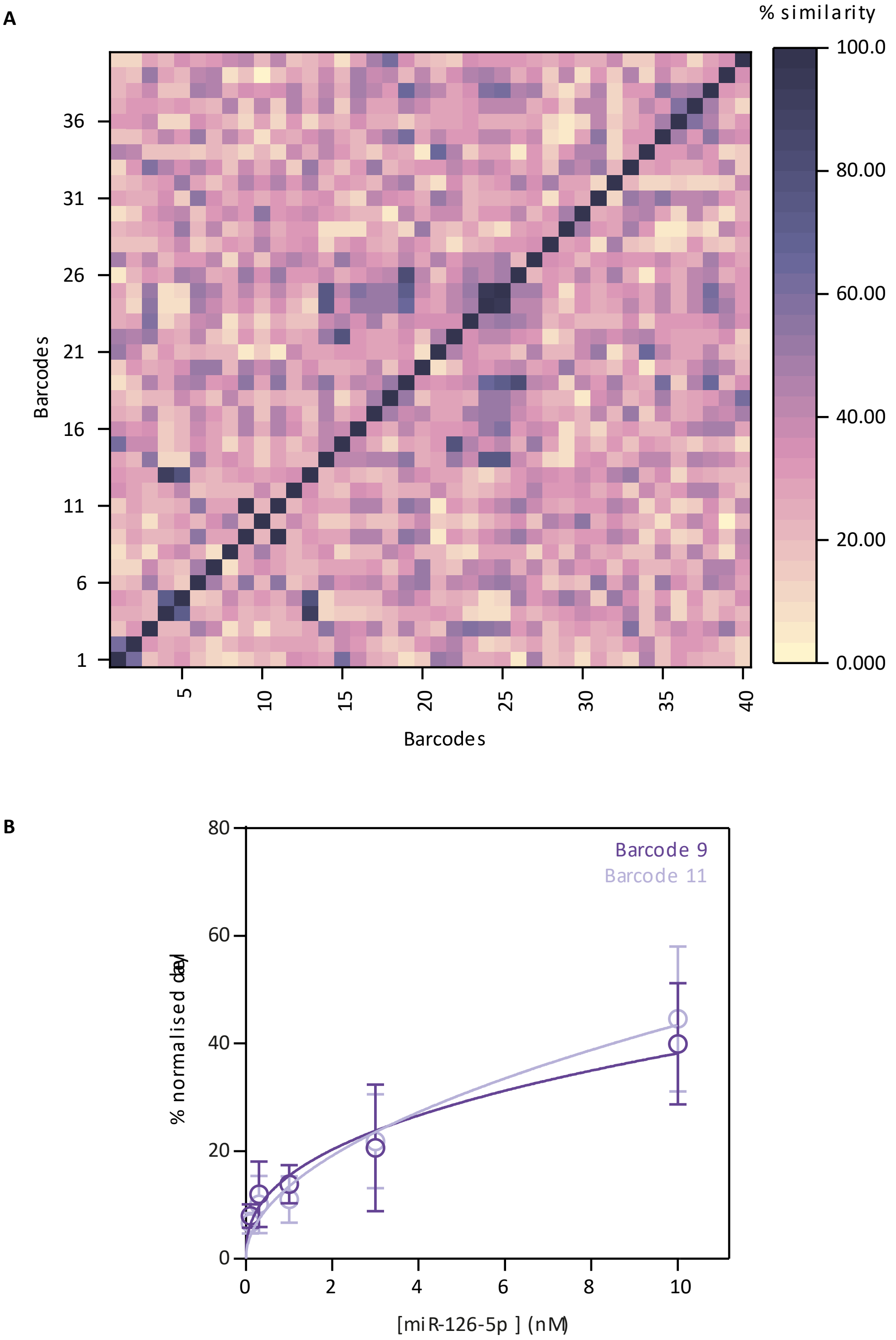


**Supplementary Fig. 4| Sequence similarity of miRNAs in multiplexed experiments.**

(A) Confusion matrix of sequence similarity of miRNAs designed to bind to barcoded probes. Two barcoded probes (barcode 9 and barcode 11) were designed to bind the same miRNA (miRNA-126-5p). Two barcoded probes (barcode 24 and barcode 25) were designed to bind miRNAs with sequence similarity ≥95 % (miRNA-18a-5p and miRNA-18b-5p). Two barcoded probes (barcoded probe 4 and barcoded probe 13) were designed to bind miRNAs with sequence similarity 95 % > X ≥ 90 % (miRNA-30d-5p and miRNA-30e-5p). (B) Concentration-percentage delay event relationship of barcodes 9 and 11 (single barcode experiments). Both barcoded probes are designed to bind miRNA-126-5p. Barcode sequence had no significant effect on the % delay (N=3, $n_{total\ events}$=17899), meaning that both probes behave the same independent of the barcode sequence. Data presented as mean ± SD.

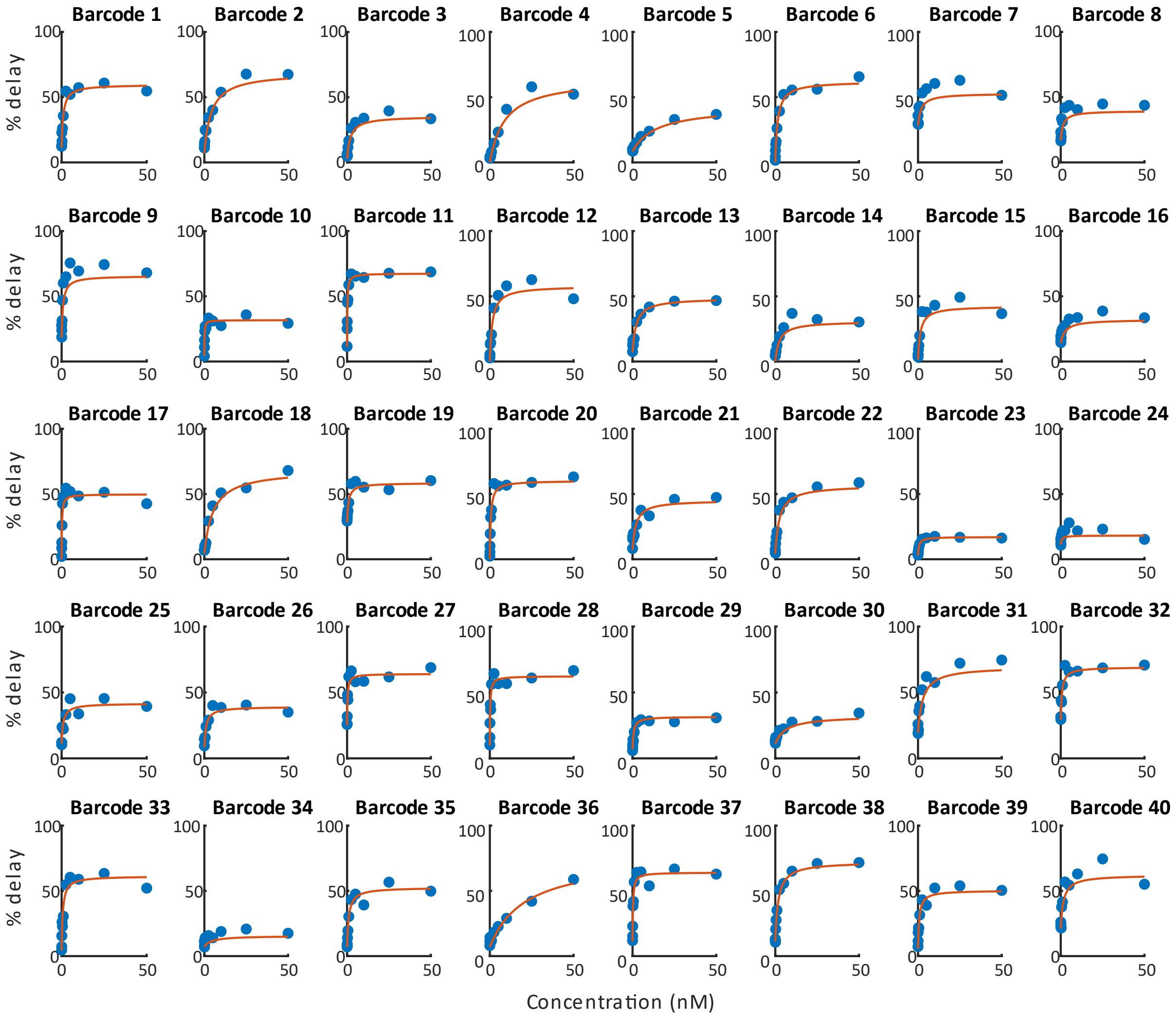


Supplementary Fig. 5| Individual barcoded probe concentration- delay relationships.

Individual miRNA / barcoded probe titration curves are plotted without background subtraction (N=5, $n_{total\ events}$=1045841).

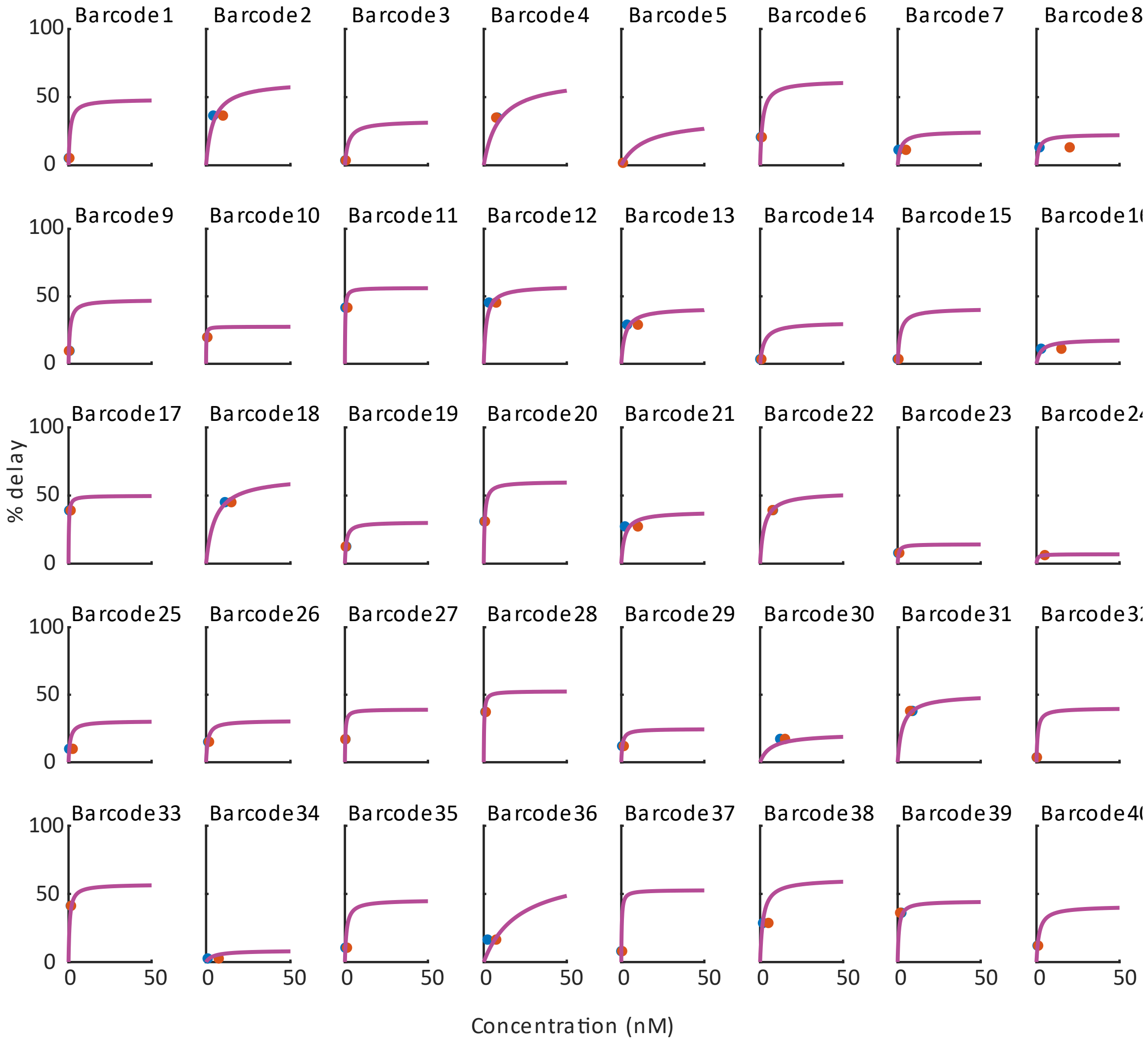


**Supplementary Fig. 6| Individual barcoded probe concentration- delay relationships.**
Individual miRNA / barcoded probe titration curves are plotted with predicted (blue) and actual (red) values as determined in Fig. 4, curves have been background subtracted using the 0 nM condition for each curve individually (N=12, $n_{\text{total events}}$=203812).

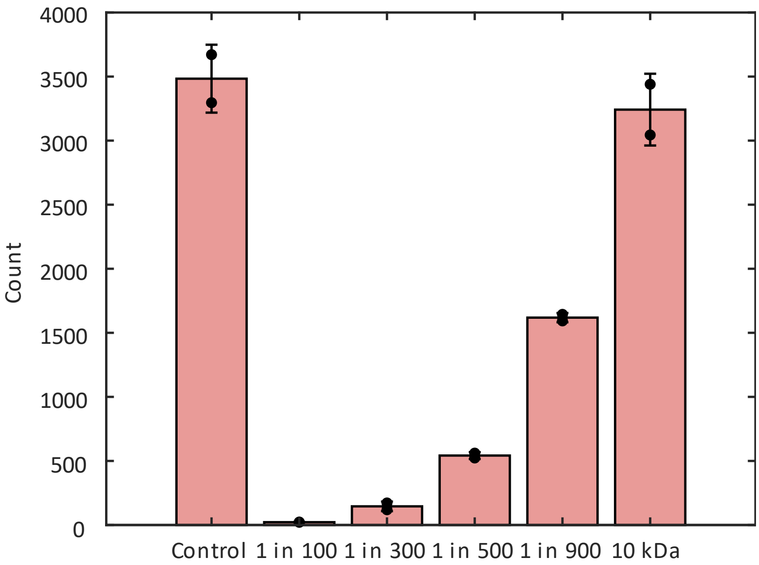


**Supplementary Fig. 7| Effect of serum on pore blockage.**

Various dilutions and conditions were tested to process serum, including: 1 in 100, 1 in 300, 1 in 500, 1 in 900 dilutions and the usage of a 10 kDa MWCO spin filter. The frequency of events increased with increasing serum dilution. When using a 10 kDa MWCO spin filter, the frequency of events was comparable to the control (barcoded probes diluted in SQB with no target), N=2, $n_{total\ events}$ = 18107. Data presented as mean ± SD.

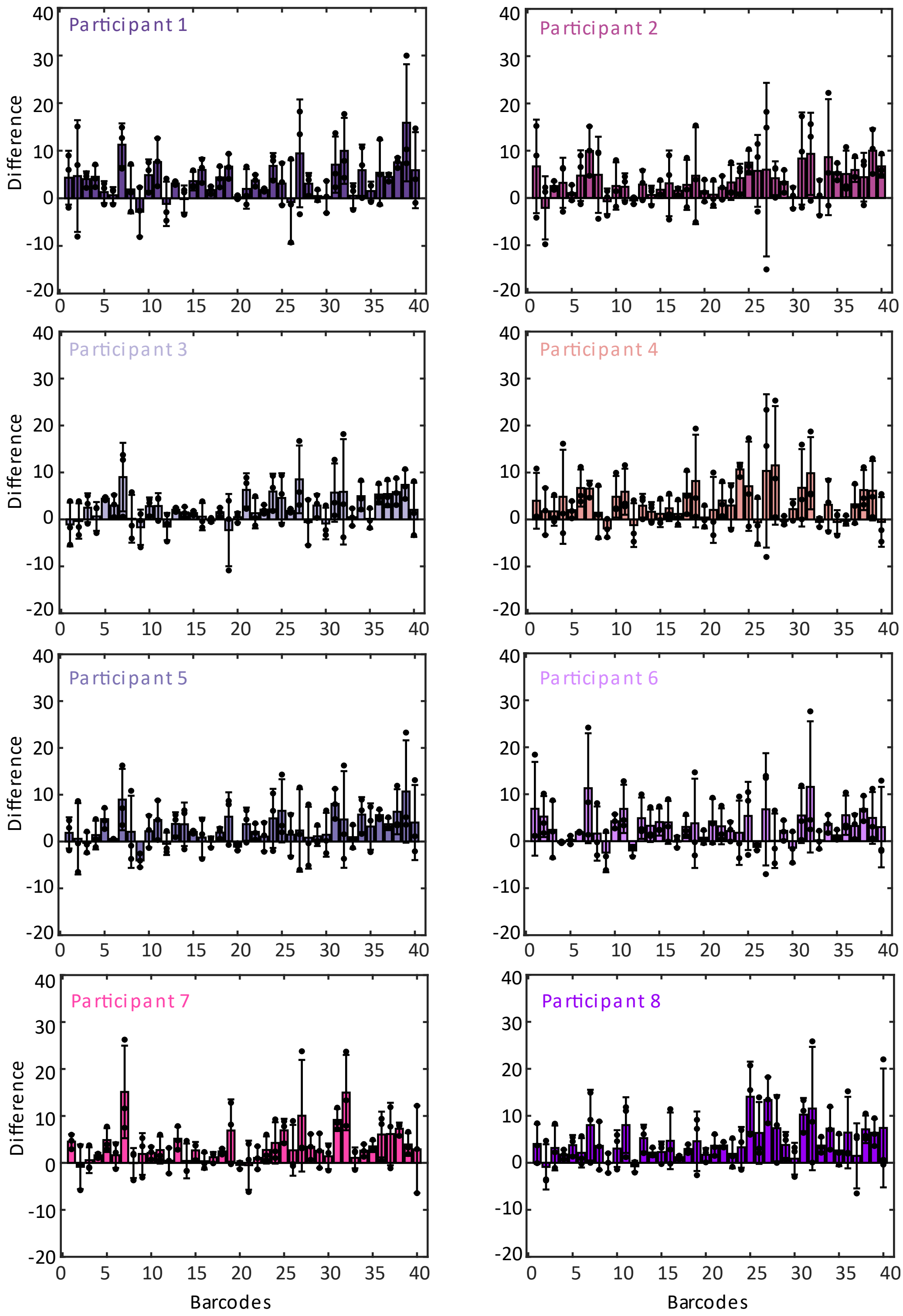


**Supplementary Fig. 8| Analysis of patient samples.**

Change in percentage delay of barcoded probes due to addition of human serum, separated by participant is shown (N=3, $n_{total\ events}$ = 194155). Data presented as mean ± SD.

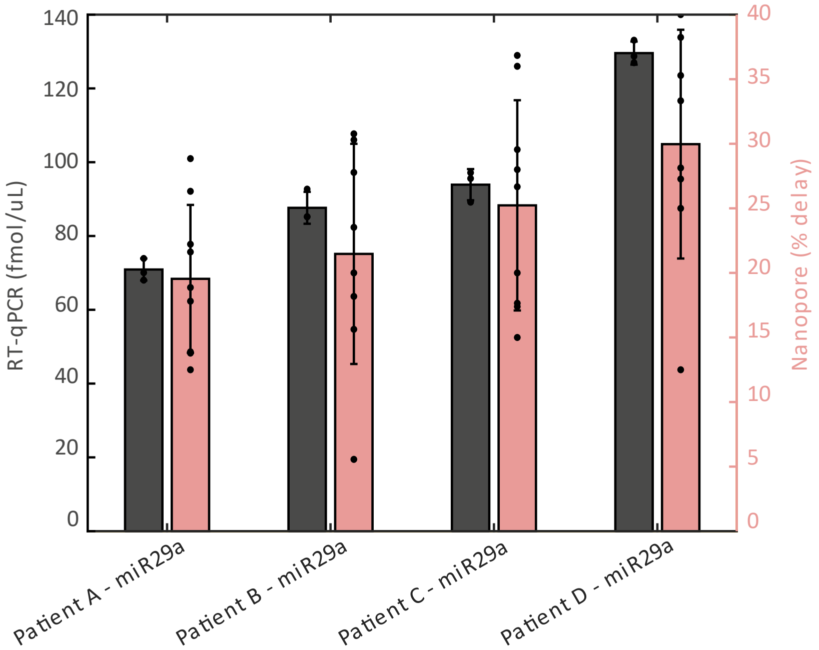


**Supplementary Fig. 9| RT-qPCR compared to nanopore experiments.**
Serum of four participants was tested with RT-qPCR for miR-29a and compared to nanopore assay, N=3, $n_{total\ events}$ = 1144. Data is presented as mean ± SD.

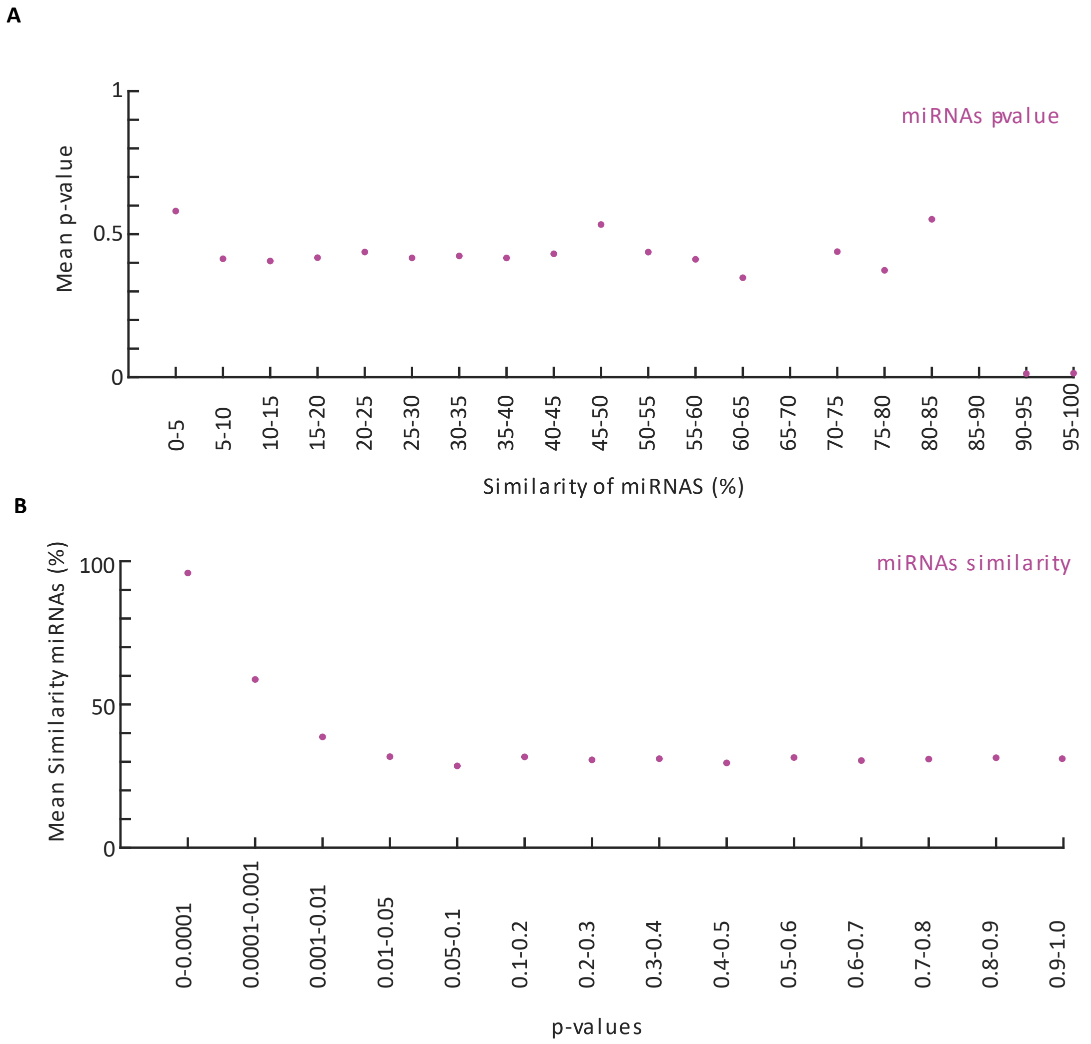


**Supplementary Fig. 10| Probe selectivity of miRNAs.**

(A) Mean P-value (One-tailed t-test), determined from change in percentage delay when single miRNAs were incubated with 40x barcoded probes, plotted against sequence similarity of miRNAs in each experimental condition (n=40). (B) Mean similarity score of miRNA sequences is inversely related to p-value (determined as above).

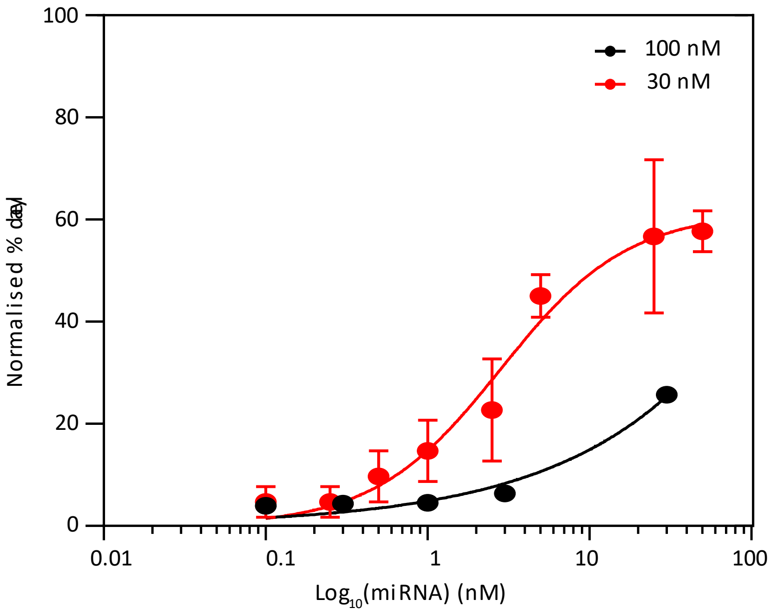


**Supplementary Fig. 11| Effect of barcoded probe concentration on assay sensitivity.** Barcoded probe #4 was prepared at two concentrations (30 nM and 100 nM). The barcoded probe was incubated with various concentrations of miR-30d-5p (0 – 50 nM) for 30 mins before loading into a flow cell. Data presented as mean ± SD. % delayed events were lower when barcoded probe concentration was higher, N=3, $n_{total\ events}$= 228406.

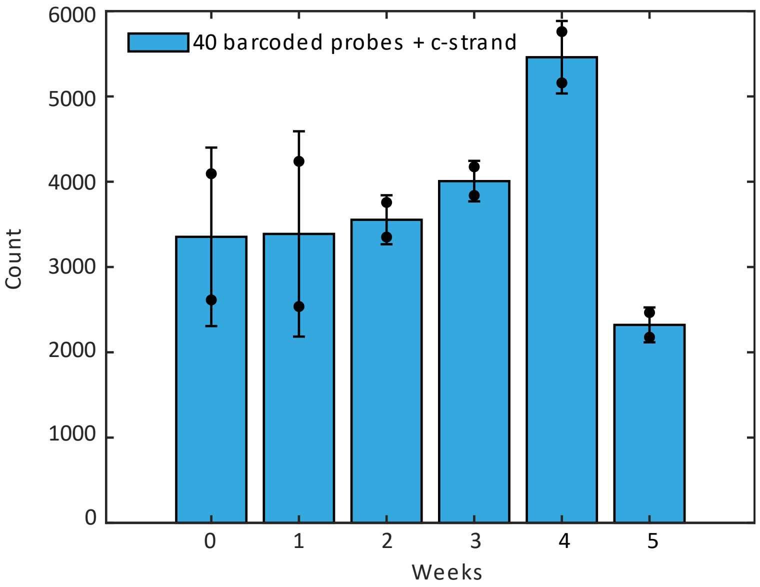


**Supplementary Fig. 12| Lifetime analysis of frozen barcoded probes.**

A mix of 40 barcoded probes was stored at -20 $^{O}$C and assessed on a weekly basis for event frequency (N=2, $n_{total\ events}$ = 99515). Event frequency was reduced after the barcoded probe mix was stored for ≥5 weeks. Data presented as mean ± SD.

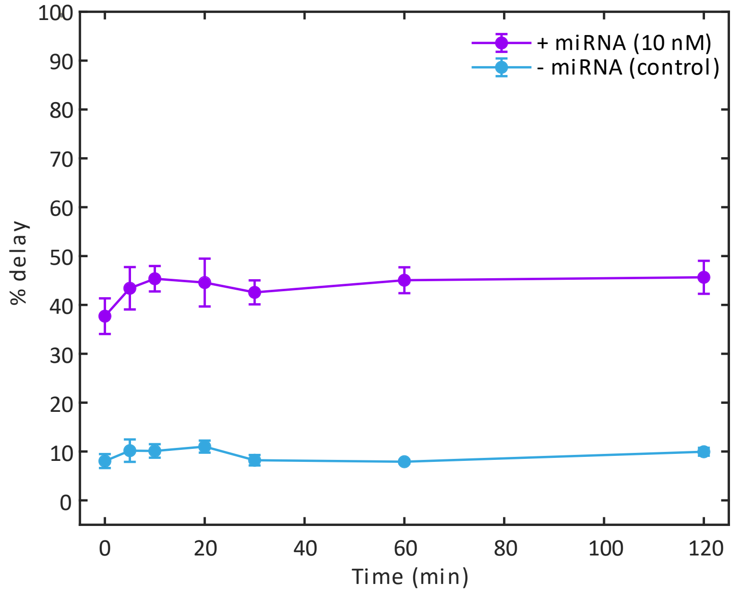


**Supplementary Fig. 13| Barcoded probe binding to miRNAs equilibrates rapidly.**

A mixture of 40 barcoded probes and 40 corresponding miRNAs (10nM) were incubated for various time periods (0 – 120 minutes) and compared to the control (0nM miRNA). The mean percentage delay plateaued after 10 minutes (N=3, $n_{totalevents}$ = 180027). Data presented as mean ± SD.

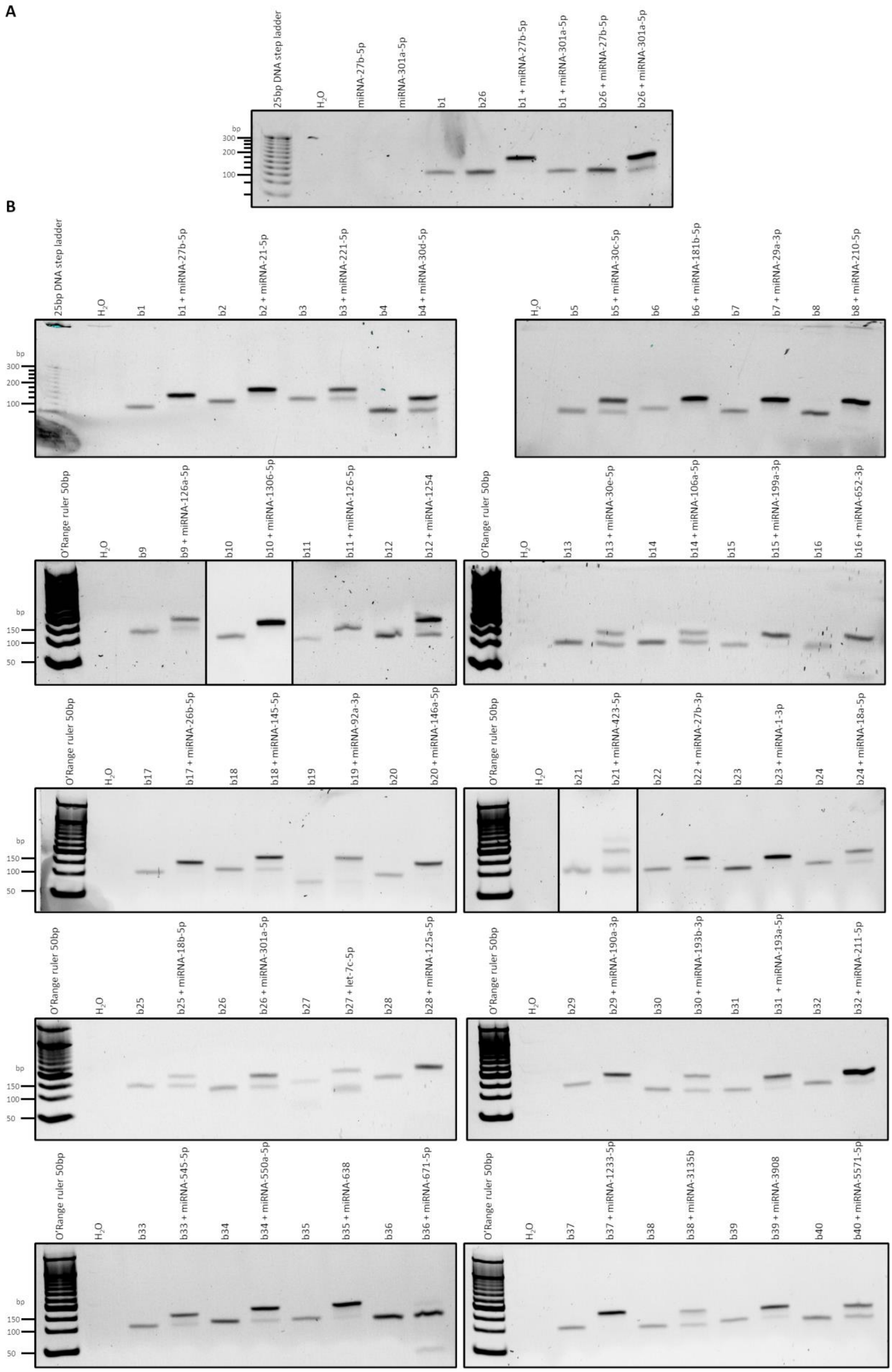
A
25bp DNA step ladder
$H_2O$
miRNA-27b-5p
miRNA-301a-5p
b1
b26
b1 + miRNA-27b-5p
b1 + miRNA-301a-5p
b26 + miRNA-27b-5p
b26 + miRNA-301a-5p
bp
300
200
100
B
25bp DNA step ladder
$H_2O$
b1
b1 + miRNA-27b-5p
b2
b2 + miRNA-21-5p
b3
b3 + miRNA-221-5p
b4
b4 + miRNA-30d-5p
$H_2O$
b5
b5 + miRNA-30c-5p
b6
b6 + miRNA-181b-5p
b7
b7 + miRNA-29a-3p
b8
b8 + miRNA-210-5p
O'Range ruler 50bp
$H_2O$
b9
b9 + miRNA-126a-5p
b10
b10 + miRNA-1306-5p
b11
b11 + miRNA-126-5p
b12
b12 + miRNA-1254
O'Range ruler 50bp
$H_2O$
b13
b13 + miRNA-30e-5p
b14
b14 + miRNA-106a-5p
b15
b15 + miRNA-199a-3p
b16
b16 + miRNA-652-3p
O'Range ruler 50bp
$H_2O$
b17
b17 + miRNA-26b-5p
b18
b18 + miRNA-145-5p
b19
b19 + miRNA-92a-3p
b20
b20 + miRNA-146a-5p
O'Range ruler 50bp
$H_2O$
b21
b21 + miRNA-423-5p
b22
b22 + miRNA-27b-3p
b23
b23 + miRNA-1-3p
b24
b24 + miRNA-18a-5p
O'Range ruler 50bp
$H_2O$
b25
b25 + miRNA-18b-5p
b26
b26 + miRNA-301a-5p
b27
b27 + let-7c-5p
b28
b28 + miRNA-125a-5p
O'Range ruler 50bp
$H_2O$
b29
b29 + miRNA-190a-3p
b30
b30 + miRNA-193b-3p
b31
b31 + miRNA-193a-5p
b32
b32 + miRNA-211-5p
O'Range ruler 50bp
$H_2O$
b33
b33 + miRNA-545-5p
b34
b34 + miRNA-550a-5p
b35
b35 + miRNA-638
b36
b36 + miRNA-671-5p
O'Range ruler 50bp
$H_2O$
b37
b37 + miRNA-1233-5p
b38
b38 + miRNA-3135b
b39
b39 + miRNA-3908
b40
b40 + miRNA-5571-5p
bp
150
100
50

**Supplementary Fig. 14| Barcoded probe binding to miRNAs- gel assay.**

Individual barcoded probes and their corresponding miRNAs were incubated for 30 mins before loading into a 15% poly-acrylamide gel, N=1. (A) Two barcodes were tested to determine barcoded probe- miRNA binding specificity. Binding was observed in the matching barcoded probe- miRNA pairs but not in the obverse condition. (B) Binding of each barcoded probe with its corresponding miRNA. Barcoded probes on their own ran at 75 – 100 bp (as expected), in the presence of their corresponding miRNA the major band was shifted by ~25 bp (attributed to the binding of miRNA, N=1).

## Supplementary methods

### Event selection pipeline

All data is presented from experiments recorded for 30 minutes with a flow cell containing a minimum of 30 active nanopores. Experiments performed which produced the data in Figure 3A are used here for illustrative purposes:
On average 36204 ± 7492 (mean ± SD) events were detected and sequenced per experiment. Application of our stated event alignment thresholds: i) sequence starts with "GGG", ii) ≥15 bases aligned to the library sequence, iii) 1 mismatched base in the first 10 bases, iv) ≤5 mismatched bases in the entire sequence, resulted in 9898 ± 3768 (mean ± SD) events per experiment. This meant an attrition rate of 73%, i.e. 73% of all sequenced events were rejected due to poor alignment with the barcode library, and 27% of all sequenced events were used in the final delay analysis. There were on average 247 ± 94 (SD) events per barcoded probe in each experiment. Our thresholding methodology was included to remove false positive events caused by basecalling, after thresholding our false positive rate in multiplexed experiments was 0.95%.

**Uncropped gel images**

Supplementary figure 14

Part A

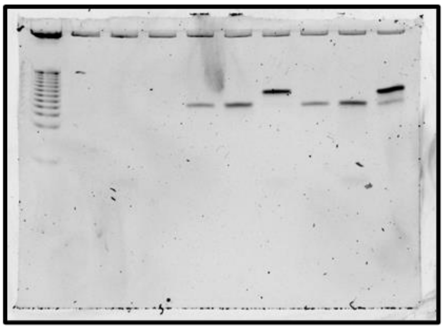

Part B

1

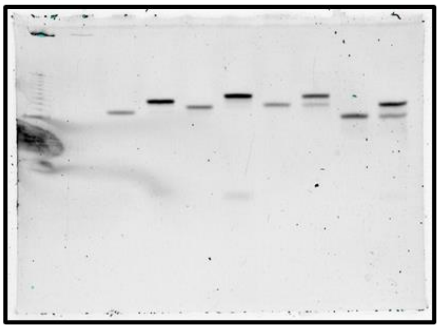

2

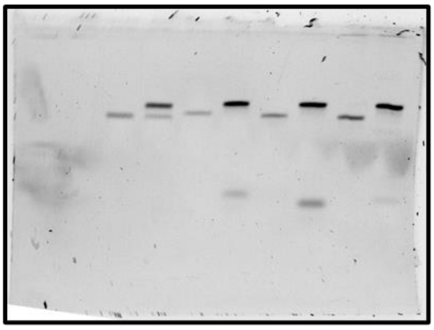

3a

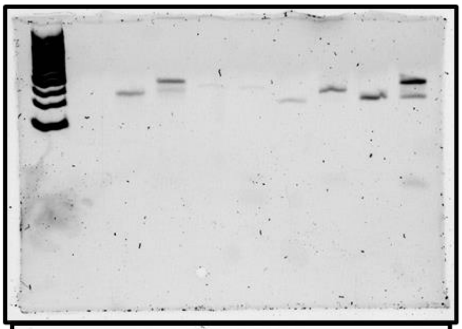

4

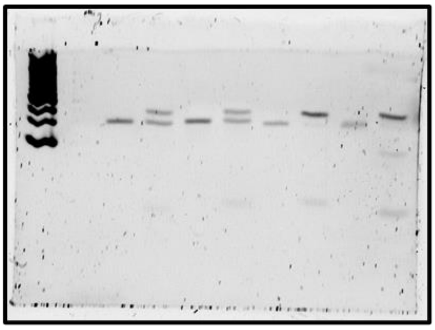

3b

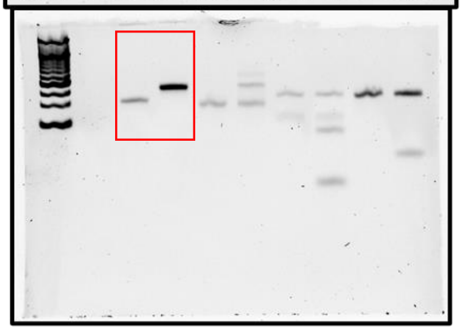

5

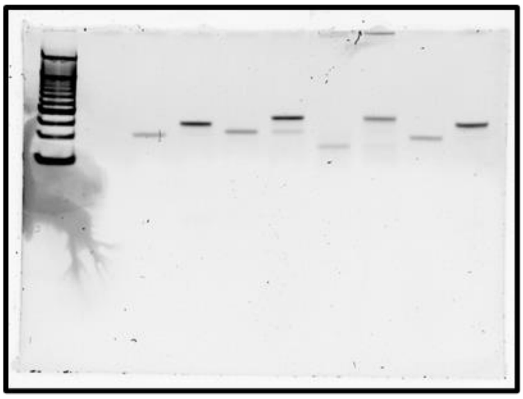

6a

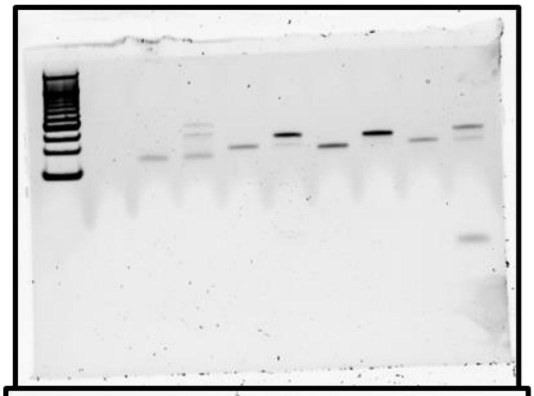

6b

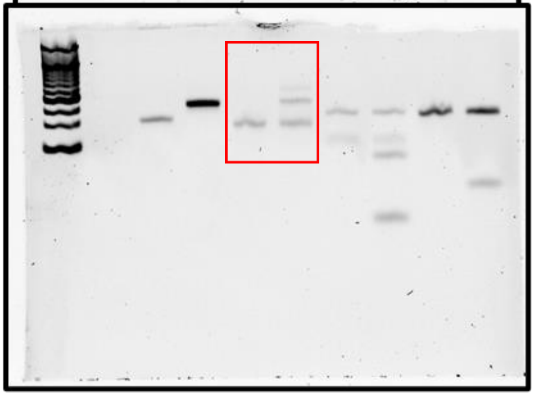

7

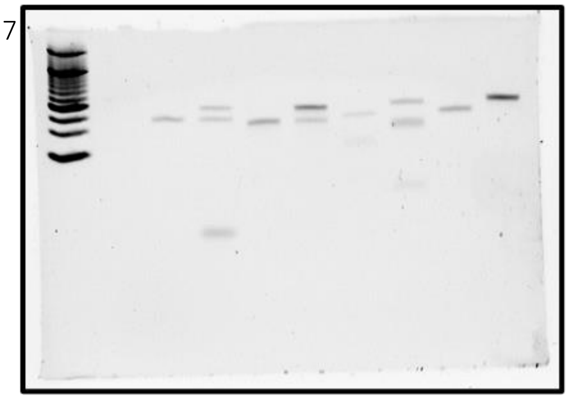

8

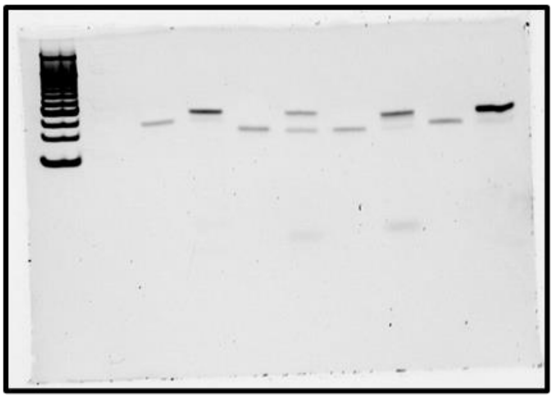

9

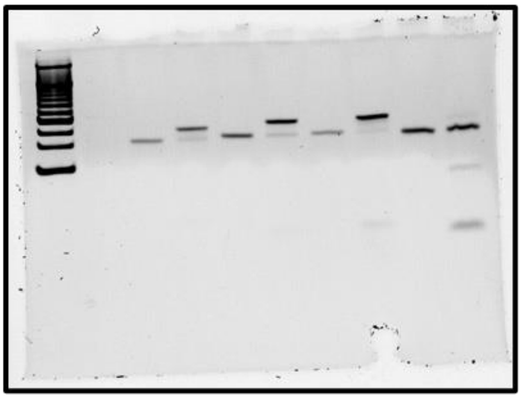

10

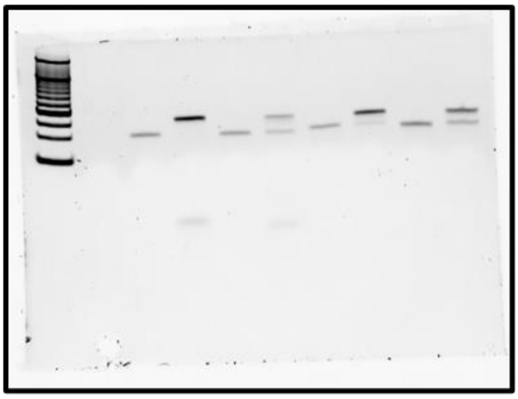

## Manual Nanopore App

The usage of the Nanopore App for data analysis is discussed in detail on the following pages.

# The Nanopore App Lite v7.19

## 1. Introduction

The Nanopore App Lite has been produced and developed by Prof Joshua B Edel, Imperial College London. It is designed to perform analysis of electrical signals arising from solid- state and biological nanopores.

The Nanopore App Lite runs within MATLAB. Analysis of large files may require the use of a GPU or server.

At the discretion of the author, the software is supplied free of charge to academic users and others working for non-commercial, non-profit making, organisations. Commercial organisations may purchase a license through written requests to the author (Joshua.edel@imperial.ac.uk).

An acknowledgement of the use of the software, in publications to which it has contributed, would be gratefully appreciated by the author.

# Contents

1. Introduction .......... 1

2. Installation .......... 3

2.1 Compatibility .......... 3

3. Launching the app .......... 3

4. Analysis of .FAST5 files from ONT sequencing devices .......... 3

4.1 Loading the file .......... 3

4.2 Identifying channel recordings for analysis .......... 4

4.3 Establishment of recording baseline and event threshold .......... 6

4.4 Event detection .......... 7

4.5 Event sequencing .......... 7

4.6 Event sequence alignment .......... 9

4.7 Event delay analysis .......... 10

# 2. Installation

The current version of The Nanopore App can be ran on the most recent release of MATLAB R2023a. MATLAB must be installed before the app can be used. MATLAB can be downloaded here: https://uk.mathworks.com/products/matlab.html.

Download 'The Nanopore App Lite' file and save locally to the hard drive.

The Nanopore App Lite is available upon request to Joshua.Edel@imperial.ac.uk

Open MATLAB. In the main MATLAB window select 'APPS' > 'Install App' > select 'The Nanopore App Lite' file from your downloads. This will install the app.

## 2.1 Compatibility

MATLAB can be ran on Linux, Windows and Mac operating systems. It is recommended that devices have >16GB RAM as a minimum memory requirement.

Raw .FAST5 files recorded within MinKNOW are often >2GB. It is therefore also important to ensure analysis computers have large capacity for storage of such files.

# 3. Launching the app

Open MATLAB

Navigate to: 'APPS' > 'The Nanopore App Lite'

> NOTE- *Click onto the app icon once to launch, depending on the speed of your device this may take a few seconds*

# 4. Analysis of .FAST5 files from ONT sequencing devices

This workflow is established for the analysis of nanopore recordings recorded with MinKNOW as .FAST5 files. It provides a pipeline to analyse multiplexed experiments which include barcoded probes, as established in Koch et al. (2023) Nature Nanotechnology.

## 4.1 . Loading the file

On the 'Import' tab within the main app window, click 'Select File(s)'

Use the file browser to find a .FAST5 file for analysis.

*NOTE: Files must be saved locally on the device*

The file will take a short while to load. It has loaded when a blue trace is visible in the bottom plot area and the file location is visible in the upper text box (Figure 1).

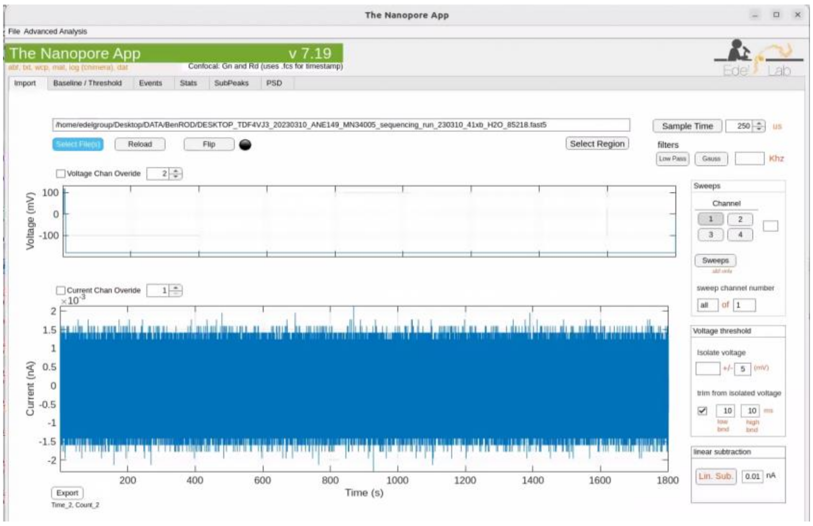


*Figure 1: Screenshot of The Nanopore App Lite with a fully loaded .FAST5 file.*

If loading a .FAST5 file which has already been basecalled in MinKNOW, it is possible to bypass sections 4.2, 4.3 and 4.4. To load files which have already been basecalled, navigate to the 'Sequence' tab within the sequencing window (Figure 2). Click the 'Basecalled (MinKNOW)' button and use the file browser to find your file for analysis.

## 4.2 Identifying channel recordings for analysis

It is possible to observe each nanopore recording individually. This is particularly useful to verify nanopore functionality as this can vary between flow cells.

Navigate to the sequencing part of the Nanopore App: 'Advanced Analysis' > 'Sequencing'. This will open a new window, this time with a blue banner (Figure 2).

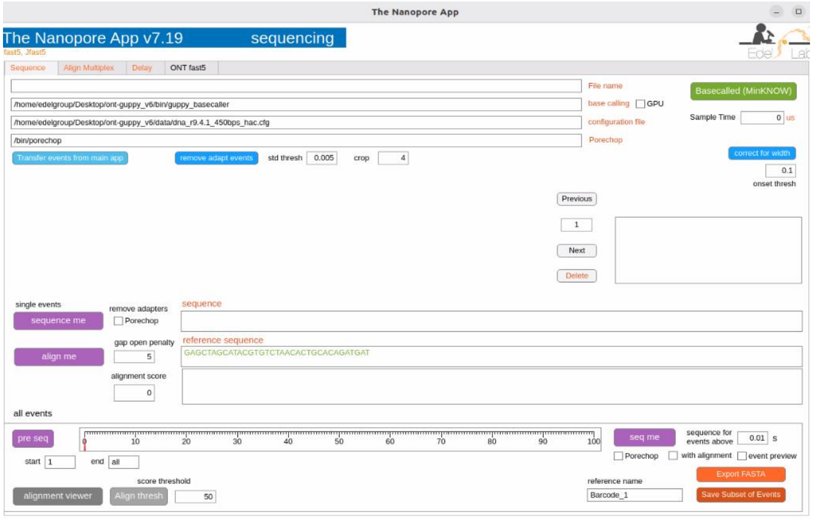


*Figure 2: Screenshot of The Nanopore App Lite sequencing window.*

In the sequencing window, switch to the 'ONT fast5' tab and begin loading channel reports. Set a range of channels (20 channels works best) and then select the 'Report' button. This will load up a further pop-up window (Figure 3).

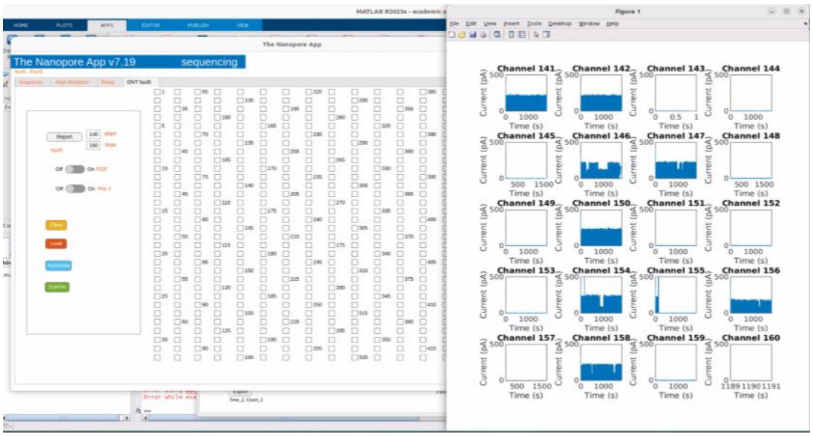


*Figure 3: Screenshot of channel reports from a .FAST5 file acquired from an ONT device.*

In the new window you can view at a glance the recordings of each nanopore separately. Channels with successful recordings in this example are: 141, 142, 146, 147, 150, 154, 156 and 158 (Figure 3).

Using the check boxes in the sequencing window, select the channel you would like to analyse and click 'Load'.

> *NOTE: Depending upon the computing power available, you can analyse multiple channels simultaneously*

Navigate back to the main window (green banner). When the channel(s) have successfully loaded, you will be able to observe the full recording(s) in the two plot windows.

Flip the channel recording by selecting the ‘Flip’ button. This process has completed when the circle changes from black to blue.

## 4.3 Establishment of recording baseline and event threshold

In the main window, navigate to the ‘Baseline/ Threshold’ tab (Figure 4).

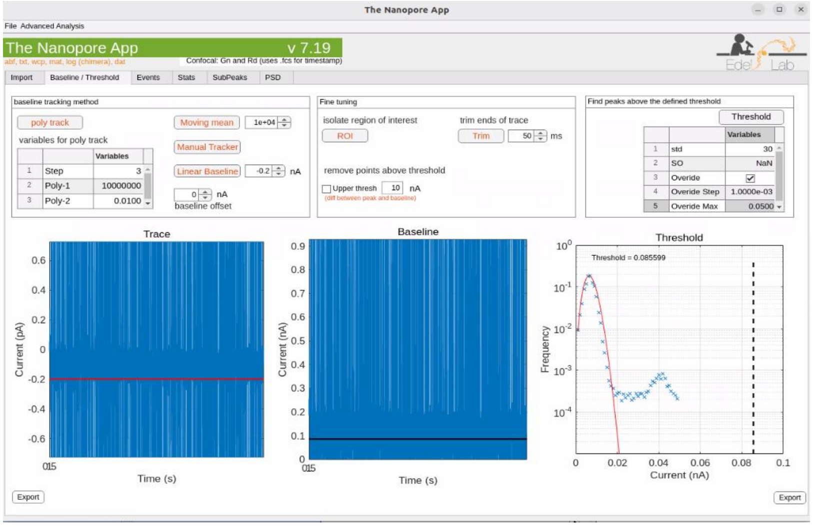


*Figure 4: Screenshot of 'Baseline / Threshold' tab with a good baseline and threshold definition.*

Set a linear baseline by inputting a value into the ‘baseline tracking method’ text box and pressing the ‘Linear Baseline’ button.

*NOTE: This is usually between -0.18 and -0.22 nA*

The baseline can be easily viewed in the left hand plot by adjusting the axis so that individual events can be observed (Figure 5).

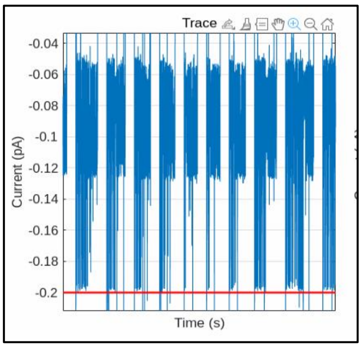


*Figure 5: Screenshot showing a linear baseline set at -0.2nA. The baseline is set just above the peak minimum event current.*

In the 'find peaks above the defined threshold' box (right hand side), define the event threshold by determining the optimum: Std (30-40), Overide Step (~0.001) and Overide Max (~0.05) (the overide check box should be ticked).

Press the 'Threshold' button to observe the fit (Poisson distribution, Figure 4). Settings are optimal when the red fit line in the right-hand plot follows the data well and the peak can be observed. It is not essential to see the entire fit for the left hand side of the plot.

## 4.4 Event detection

In the main window, switch to the 'Events' tab.

Press the 'Find Peaks' button (neither the 'FWHM' or 'Buffer +/- bins' boxes should be checked. When this process is complete, the event trace will appear in the top plot area (Figure 6). This function will find all events as defined by the thresholds previously established. Events will be marked in red.

Further thresholds to events can be applied using the 'Thresholds' box e.g. events must have a duration >0.1s (Figure 6). Press the 'Thresholds'  button to apply these options.

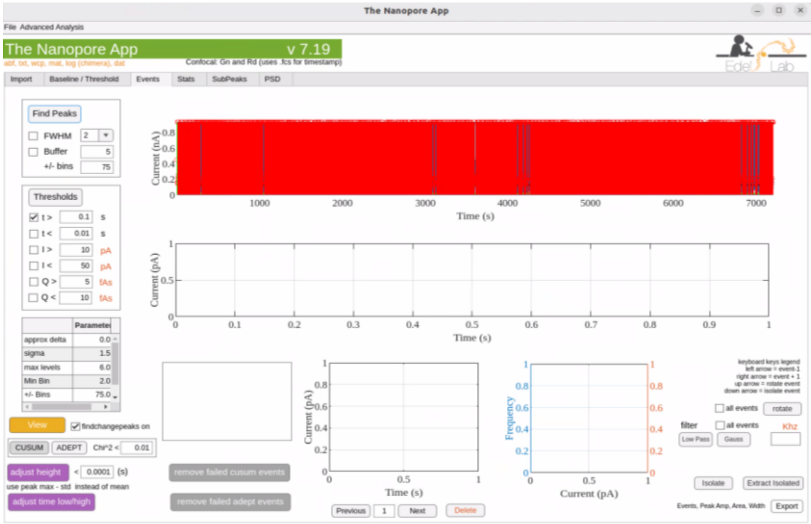


*Figure 6: Screenshot showing event detection using Find peaks and then applying a threshold of event duration >0.1s.*

## 4.5 Event sequencing

Switch to the sequencing window and navigate to the 'Sequence' tab.

Ensure that the file locations for the base calling and configuration files are correct. If using a GPU for analysis check this box too.

> *NOTE- These files are provided by Oxford Nanopore Technologies and can be changed depending upon your application. In this example we use the 'guppy basecaller' and '450bps*

*high accuracy calling model' files. Basecallers can be downloaded from the ONT Github site (https://github.com/nanoporetech)*

Press the 'Transfer events from main app' button. This will load each individual event.

You can navigate between each event using the 'previous' and 'next' buttons.

Press the 'remove adapter events' button to remove adapter-only signals.

Press the 'correct for width' button to identify the start of an event. The event onset threshold can be adjusted using the associated text box. This process is complete when a green broken line is present at the onset of an event in the left hand side plot (Figure 7).

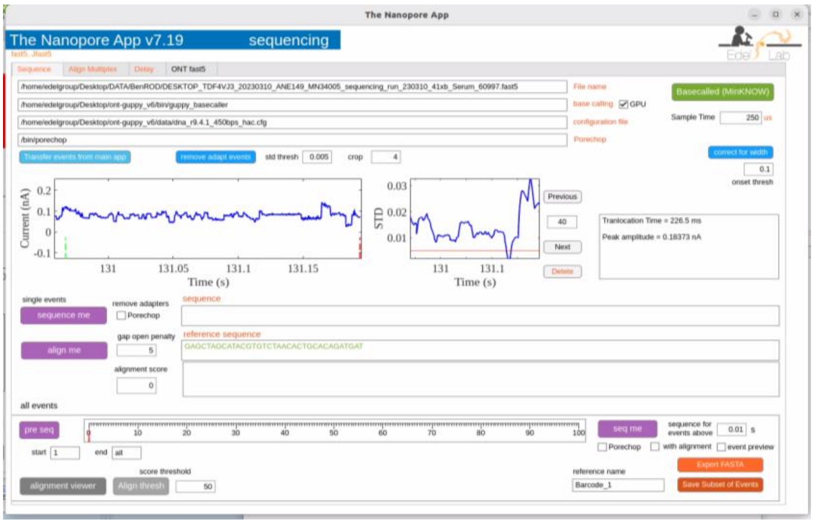


*Figure 7: Screenshot of event sequencing tab, with events corrected for width.*

To complete sequencing and base calling of events, first the file must be pre-sequenced by pressing the 'pre-seq' button. The progress bar indicates that this process is occurring. Depending on the number of events to be sequenced and the computing power available, this could take some time (it is the longest processing step in this pipeline). Finally the pre-sequenced files can be sequenced by pressing the 'seq me' button. The process is complete when you can observe the event base sequence below the plot area (Figure 8).

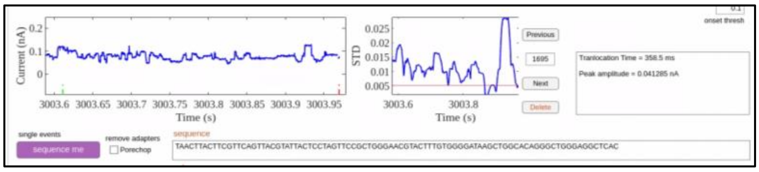


*Figure 8: Screenshot of event trace and event sequence.*

At this point the events can be saved as a .FASTA file, this is advantageous as it significantly reduces the file size and means sequencing does not need to be repeated in the future. The file name can be set using the 'reference name' text box.

*NOTE- to open .FASTA files in the future, use the 'Basecalled (MinKNOW)' button (green) which is situated in the top right of the 'Sequence' tab in the sequencing window*

## 4.6 Event sequence alignment

Switch to the 'Align Multiplex' tab within the sequencing window.

Input the barcode library sequences into the main text box. Each sequence should be separated by a blank line. There is no limit to the number of barcode sequences which can be included here.

Each event can be aligned against the barcode library by using an alignment scoring method. Parameters can be altered in the 'Alignment' box. Parameters are: Gap penalty, base match score, sequence length is less than *x* bases and base mismatch score. Events can be aligned with the 'align me' or 'Norm. align' buttons (Figure 9). The normalised alignment button normalises scores for barcodes of different lengths.

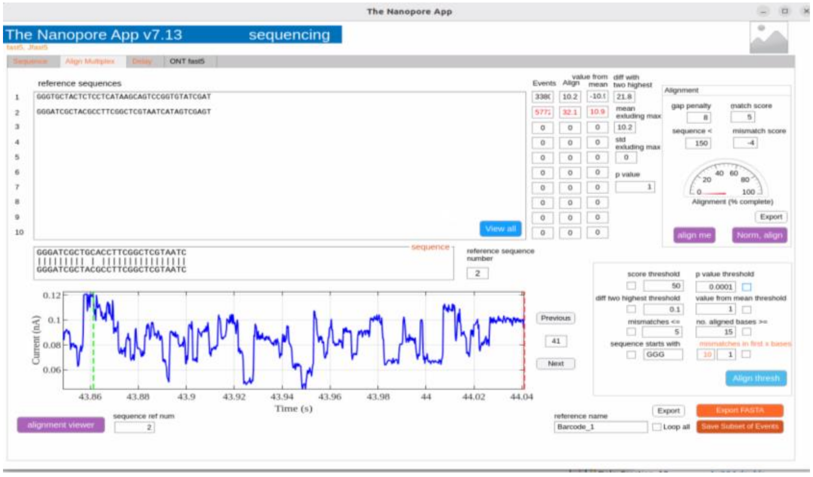


*Figure 9: Screenshot of event alignment window. Event shown has been aligned to library sequence #2.*

Alignment quality thresholds can be applied to filter out poorly resolved events. To activate a parameter, tick the associate check- box and input a value. Press the 'Align thresh' button to apply the thresholds (to reset the event alignment or remove thresholds, press the 'align me' or 'Norm. align' button).

*NOTE- The thresholding parameters should be optimised for each application.*

You can export alignment statistics by pressing the 'Export' button in the 'Alignment' box.

Events can be exported as a .FASTA file by pressing the ‘export FASTA’ button. The file name can be set using the ‘reference name’ text box.

## 4.7 Event delay analysis

To distinguish between barcode only and analyte- bound events, delay analysis can be performed.

Navigate to the ‘Delay’ tab within the sequencing window.

Currently there are two delay detection algorithms available within the app: Moving mean and Spectral entropy. The moving mean method converts an each event signal to a moving standard deviation, when the moving standard deviation drops below a certain threshold for a specified duration the event is classified as delayed. The spectral entropy of a signal is a measure of its power distribution and is derived from Shannon entropy. It reduces the dimensionality of the time series by encoding the spectral features of the signal. Both transformations of the event signal can be viewed in the right hand side plots (Figure 10). These algorithms will divide each event population into two sub-groups ‘no delay’ and ‘delay’.

To choose a delay detection algorithm, select it in the ‘Delay Algorithm’ box.

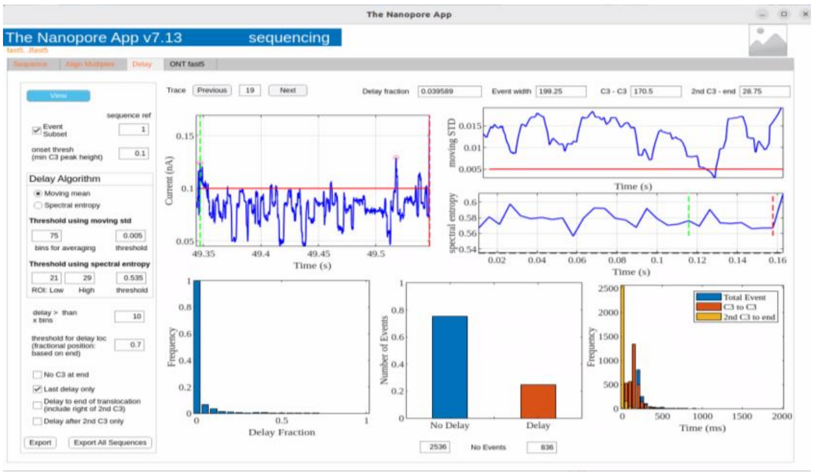


*Figure 10: Screenshot showing a delayed event using the moving mean algorithm.*

The parameters for both algorithms can be altered using the delay algorithm box.

The moving mean can be optimised by changing the number of ‘bins for averaging’ and the ‘threshold’ value. The ‘bins for averaging’ determines how many bins the event signal is split up into. The ‘threshold’ value is a value at which the moving standard deviation is determined to be low.

The spectral entropy can be optimised by changing the definition of the ROI within the transformation (denoted by broken green and red lines), using the ‘Low’ and ‘High’ boxes . The ‘threshold’ value is a value at which the spectral entropy is determined to be low.

Altering the ‘delay > than *x* bins’ will adjust both algorithms by changing the period through which the moving standard deviation or spectral entropy will have to be below the threshold in order to be classified as delayed.

The ‘threshold for delay location’ and 4x check boxes below can be used to further tune the algorithm by altering where in the event signal the delay period is detected.

By ticking the event subset check-box and indicating the ‘sequence reference’ number, users can view delay statistics for each event sorted by library sequence. All statistics can be exported for the current sequence reference by pressing the ‘Export’ button whereas all statistics for all library sequences can be exported by pressing the ‘Export All Sequences’ button.
